# The tegument protein VP22 of pseudorabies virus inhibits cGAS condensation by inducing nuclear-to-cytoplasmic translocation of DDX21

**DOI:** 10.1371/journal.ppat.1013549

**Published:** 2025-09-29

**Authors:** Kesen Liu, Wandi Cao, Hanhua Zhang, Xingmiao Yang, Chengyue Wu, Xingya Wang, Qian Sun, Jianxiong Guo, Ting Zhang, Yan Liu, Xing Liu

**Affiliations:** 1 Key Laboratory of Animal Diseases Diagnostic and Immunology, Ministry of Agriculture, MOE International Joint Collaborative Research Laboratory for Animal Health & Food Safety, College of Veterinary Medicine, Nanjing Agricultural University, Nanjing, China; 2 Guangdong Provincial Key Laboratory of Infection Immunity and Inflammation, Department of Pathogen Biology, Shenzhen University Medical School, Shenzhen, China; 3 Changchun Veterinary Research Institute, Chinese Academy of Agricultural Sciences, Changchun, China; 4 Shenzhen Health Administrative Center for Cadre and Talent, Shenzhen, China; State University of New York Upstate Medical University, UNITED STATES OF AMERICA

## Abstract

Cyclic GMP-AMP synthase (cGAS) is a pivotal DNA sensor that initiates antiviral responses, yet the mechanisms by which viruses evade cGAS-mediated innate immunity remain poorly understood. Here, we identified VP22, a tegument protein of pseudorabies virus (PRV), a member of the Alphaherpesvirinae subfamily, as a viral antagonist of the type I interferon (IFN-I) response through hijacking the host RNA helicase DDX21. Specifically, VP22 impairs 2’3’-cyclic GMP–AMP (cGAMP) synthesis by disrupting cGAS condensation. In vivo, cGAS restricts the replication of VP22-deficient PRV and attenuates its pathogenicity, an effect neutralized by VP22. Notably, DDX21 is essential for VP22-mediated inhibition of cGAS activity. Mechanistically, VP22 stabilizes DDX21 protein level and enhances its interaction with cGAS. Furthermore, VP22 promotes the translocation of DDX21 from the nucleus to the cytoplasm, a process required for inhibition of cGAS condensation and activation. Collectively, these findings reveal a previously unrecognized, host-dependent mechanism by which PRV subverts cGAS signaling, shedding light on viral strategies to subvert host DNA sensing and innate immunity.

## Author summary

Viruses and their hosts are engaged in a constant evolutionary arms race, with viruses developing diverse strategies to evade immune surveillance and establish persistent infections. In this study, we identified a novel immune evasion mechanism in which VP22, a tegument protein of pseudorabies virus (PRV) that is an alphaherpesvirus, suppresses activation of the DNA sensor cGAS by hijacking the host RNA helicase DDX21. Specifically, VP22 stabilizes DDX21 by preventing its degradation through the ubiquitin–proteasome pathway, thereby enhancing its interaction with cGAS. Moreover, VP22 promotes the cytoplasmic translocation of DDX21, a step required for VP22-mediated suppression of cGAS activation. These findings reveal a previously unrecognized strategy by which a herpesvirus manipulates a host helicase to subvert cGAS-mediated DNA sensing and innate immunity, offering new insights into viral immune evasion and potential targets for antiviral intervention.

## Introduction

The innate immune system defends against viral infections through pattern recognition receptors (PRRs) that detect pathogen-associated molecular patterns (PAMPs). Among these, cyclic GMP–AMP synthase (cGAS) is a key cytosolic DNA sensor that recognizes double-stranded DNA (dsDNA) and plays a crucial role in mounting antiviral responses against DNA viruses [1–3]. Upon binding dsDNA, cGAS undergoes oligomerization and liquid–liquid phase separation (LLPS), both essential for its activation. Activated cGAS catalyzes the synthesis of 2′3′-cyclic GMP–AMP (cGAMP) from ATP and GTP [4–6]. cGAMP then binds to and activates the adaptor protein Stimulator of Interferon Genes (STING), initiating downstream signaling cascades that ultimately lead to the production of type I interferons (IFN-I) [7,8]. To evade this antiviral surveillance, many DNA viruses have evolved sophisticated strategies to disrupt cGAS signaling. For example, Herpes Simplex Virus 1(HSV-1) triggers inflammasome activation and caspase-1 cleavage, whereas Vaccinia Virus (VACV) similarly engages these pathways [9], resulting in the proteolytic processing of cGAS [10]. Moreover, HSV-1 diminishes cGAS activity through multiple host-directed mechanisms: it modulates the host protein PCBP2 to suppress cGAS LLPS upon DNA binding [11], and alters PARP1 phosphorylation to promote its cytoplasmic translocation, where it binds cGAS and inhibits its DNA recognition capability [12]. These findings underscore the complexity and diversity of viral strategies to evade innate DNA sensing, reflecting an ongoing evolutionary arms race between host defenses and viral countermeasures.

The DEAD-box (DDX) family of RNA helicases comprises evolutionarily conserved proteins characterized by the signature DEAD (Asp-Glu-Ala-Asp) motif. These helicases play critical roles in various aspects of RNA metabolism, including transcription, splicing, ribosome biogenesis, RNA transport, and degradation [13,14]. In recent years, several DDX helicases have emerged as important regulators of innate immunity, particularly in modulating antiviral signaling pathways. For example, DDX41 functions as a cytosolic DNA sensor that activates STING-dependent type I interferon (IFN-I) responses [15], while DDX3 acts as a co-factor in RIG-I-like receptor (RLR) signaling, enhancing IFN-β production during RNA virus infection [16]. DDX21 has recently gained attention for its potential role in antiviral immunity. Emerging evidence suggests that DDX21 shuttles between nucleus and cytoplasm, especially under stress or viral infection, indicating a broader regulatory role in host defense. During influenza virus infection, DDX21 translocates from the nucleolus to the cytoplasm, where it inhibits IFN-β production [16]. Moreover, as part of the DDX1–DDX21–DHX36 complex, DDX21 contributes to the recognition of viral double-stranded RNA and activates NF-κB and IRF3 signaling pathways [17–19]. Despite these insights, the role of DDX21 in DNA-triggered innate immunity—particularly its potential involvement in the cGAS–STING pathway—remains largely uncharacterized.

Pseudorabies virus (PRV), a double-stranded DNA virus belonging to the *Alphaherpesvirinae* subfamily, is the causative agent of Aujeszky’s disease in swine and can infect a wide range of mammalian species [20]. Emerging evidence suggests that PRV, in very rare cases, may infect humans, indicating its potential for zoonotic transmission [20,21]. Like other herpesviruses, PRV encodes numerous tegument proteins that play critical roles in modulating early infection events and evading host immune defenses. Among these, VP22, a conserved tegument protein encoded by the UL49 gene, is one of the most abundant components of the virion and plays critical roles in viral assembly and maturation. It dynamically localizes between the cytoplasm and nucleus and, through interactions with the host cytoskeleton and chromatin, facilitates protein transport and regulates viral gene transcription [22–24]. While HSV-1 VP22 has been shown to promote viral gene expression and inhibit cGAS-mediated DNA sensing [25–28], the exact mechanism by which PRV VP22 modulates DNA sensing has remained unclear. In this study, we demonstrated that PRV VP22 inhibits cGAS activation by inducing the nuclear-to-cytoplasmic translocation of DDX21 and enhancing its interaction with cGAS, thereby antagonizing cGAS condensation and liquid droplet formation. These findings reveal a novel host-dependent strategy by which PRV impairs cGAS-triggered innate immune signaling.

## Results

### PRV VP22 inhibits cGAS-STING mediated type I interferon pathway

**Fig 1:**
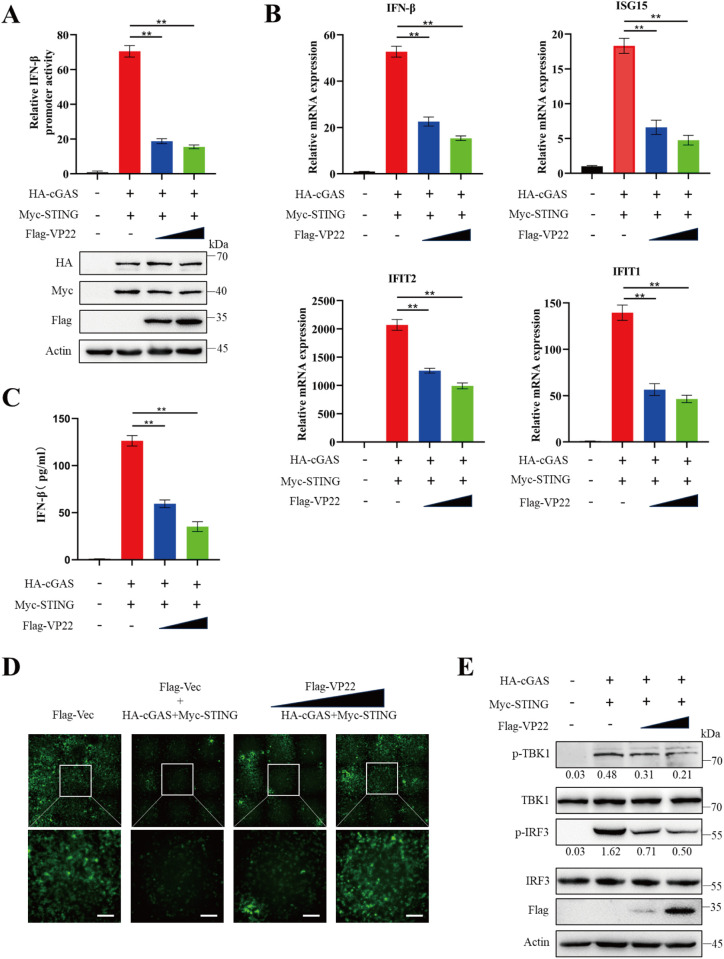
PRV VP22 inhibits the cGAS–STING–mediated type I interferon pathway. (A) HEK-293T cells seeded in 24-well plates were co-transfected with IFN-β-Luc, pRL-TK, HA-cGAS, or Myc-STING along with increasing amounts of Flag-VP22. At 24 hours post-transfection (hpt), cells were lysed for luciferase reporter assays (upper panel) and immunoblotting (lower panels) with antibodies against HA, Flag, and β-actin. (B) HEK-293T cells were treated as in panel A, but without IFN-β-Luc and pRL-TK transfection. Total RNA was extracted and subjected to RT-qPCR analysis for *IFN-β*, *ISG15*, *IFIT2*, *IFIT1*, and *18S rRNA* mRNA expression. (C) HEK-293T cells were treated as in panel B. Cell culture supernatants were collected and analyzed for IFN-β production using an IFN-β ELISA kit. (D) Supernatants from panel C were incubated with fresh confluent HEK-293T cells. After 24 h, the cells were infected with VSV-GFP at an MOI of 0.01. At 24 hpi, GFP fluorescence was visualized by microscopy. Scale bars: 100 μm. (E) HEK-293T cells were treated as in panel B. Cell lysates were harvested and analyzed by western blot using antibodies against Flag, IRF3, phosphorylated IRF3 (p-IRF3), TBK1, phosphorylated TBK1 (p-TBK1), and β-actin. Data are representative of at least three independent experiments with similar results (mean ± SD of n = 3 biological replicates in panels A–C). ** P < 0.01.

VP22, a viral tegument protein encoded by the UL49 gene and conserved among alpha-herpesviruses, shares only 26% sequence homology between PRV and HSV [29]. Previous studies have shown that HSV VP22 suppresses cGAS-mediated innate immune responses [25]. To investigate whether PRV VP22 similarly modulates cGAS signaling, we first performed a dual-luciferase reporter (DLR) assay and found that PRV VP22 significantly inhibited cGAS–STING-induced IFN-β promoter activation in HEK-293T cells (Fig 1A). In line with this finding, mRNA levels of *IFN-β*, *ISG15*, *IFIT2*, and *IFIT1* were markedly reduced in cells co-transfected with VP22 and cGAS–STING, compared to controls (Fig 1B). Furthermore, supernatants from cells expressing both VP22 and cGAS-STING exhibited decreased IFN-β levels and failed to restrict VSV-GFP replication, as evidenced by sustained GFP fluorescence (Fig 1C and 1D), suggesting that VP22 impairs the secretion of IFN-β or antiviral factors typically induced by cGAS-STING activation. Since phosphorylation of TBK1 and IRF3 is a hallmark of cGAS-STING pathway activation, we next investigated whether VP22 interferes with this process. Western blot analysis revealed that VP22 overexpression substantially suppressed TBK1 and IRF3 phosphorylation in response to cGAS–STING stimulation (Fig 1E). Collectively, these results demonstrate that PRV VP22 inhibits cGAS–STING signaling.

### PRV VP22 interacts with cGAS and antagonize its enzyme activity

**Fig 2:**
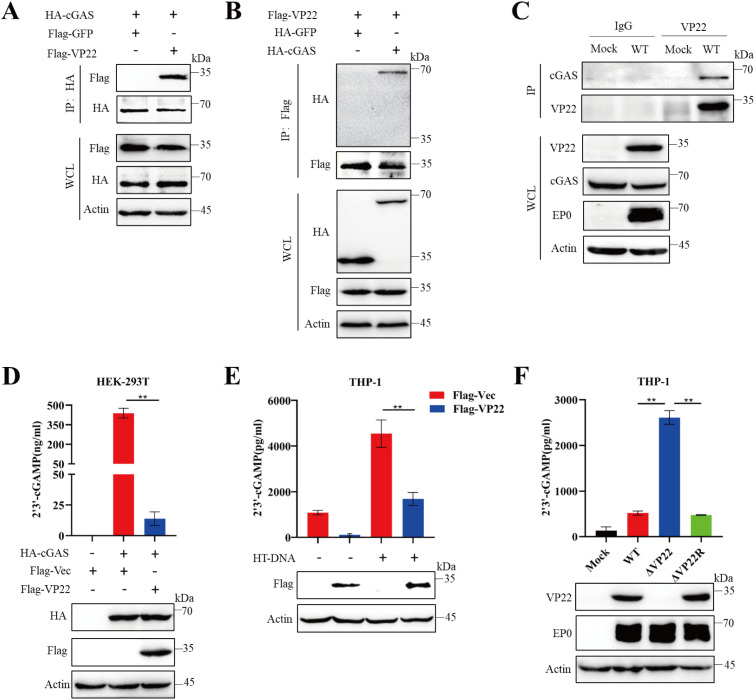
PRV VP22 interacts with cGAS and inhibits its enzymatic activity. (A) HEK-293T cells seeded in 6-cm dishes were co-transfected with Flag-VP22 and the indicated plasmids (HA-GFP or HA-cGAS). At 24 hours post-transfection (hpt), cells were subjected to immunoprecipitation (IP) using anti-Flag magnetic beads. Whole-cell lysates (WCLs) and immunoprecipitates were analyzed by immunoblotting with antibodies against HA, Flag, and β-actin. (B) HEK-293T cells seeded in 6-cm dishes were transfected with HA-cGAS along with the indicated plasmids (Flag-GFP or Flag-VP22). At 24 hpt, cells were processed for IP as in panel A. (C) THP-1 cells seeded in 10-cm dishes were either mock-infected or infected with PRV. At 24 hours post-infection (hpi), cell lysates were immunoprecipitated with a mouse anti-VP22 antibody or control IgG, followed by western blot analysis with against cGAS, VP22, EP0 and β-actin. (D) HEK-293T cells were transfected with either an empty vector or Flag-VP22 for 24 h, after which cell lysates were harvested to quantify cGAMP levels using an ELISA kit. (E) THP-1 cells were infected with a lentivirus expressing either an empty vector or Flag-VP22 for 48 h, followed by transfection with HT-DNA (2 μg/mL) for 6 h. cGAMP levels were measured as in panel D. (F) THP-1 cells were mock-infected or infected with PRV-WT, ΔVP22, or ΔVP22R at an MOI of 1 for 12 h. cGAMP levels were determined as in panel D. Data are representative of at least three independent experiments with similar results (mean ± SD of n = 3 biological replicates in panels D–F). ** P < 0.01.

Given that VP22 suppresses cGAS-mediated antiviral signaling, we hypothesized that PRV VP22, like HSV-1 VP22, antagonizes cGAS activity [25]. To test this, we first examined the interaction between PRV VP22 and cGAS. Using a co-immunoprecipitation (Co-IP) assay, we found that VP22 specifically associates with cGAS, but not with the control GFP protein (Fig 2A and 2B). To determine whether this interaction occurs under physiological conditions, we performed Co-IP in PRV-infected cells and observed a clear association between VP22 and endogenous cGAS (Fig 2C), indicating that VP22 forms a complex with cGAS during infection.

As the enzymatic product of cGAS, cGAMP functions as a second messenger that activates downstream immune responses, thus quantifying cGAMP levels is essential for assessing cGAS activity. HEK-293T cells were transfected with cGAS plasmids along with either VP22 or a control vector, and cGAMP levels were measured using an ELISA kit. As expected, VP22 overexpression significantly reduced cGAMP production (Fig 2D). Similarly, in THP-1 cells stimulated with herring testis (HT) DNA to activate cGAS, VP22 overexpression led to a marked decrease in cGAMP synthesis compared to control cells (Fig 2E). Furthermore, THP-1 cells infected with a VP22-deficient mutant PRV (ΔVP22) produced significantly higher levels of cGAMP than those infected with wild-type PRV (WT) or a VP22-reconstituted virus (ΔVP22R), reinforcing the functional role of VP22 in suppressing cGAS activity during infection (Fig 2F). Taken together, these results demonstrate that PRV VP22 interacts with cGAS and inhibits its enzymatic activity by suppressing the synthesis of cGAMP.

### PRV VP22 antagonizes cGAS condensation

**Fig 3:**
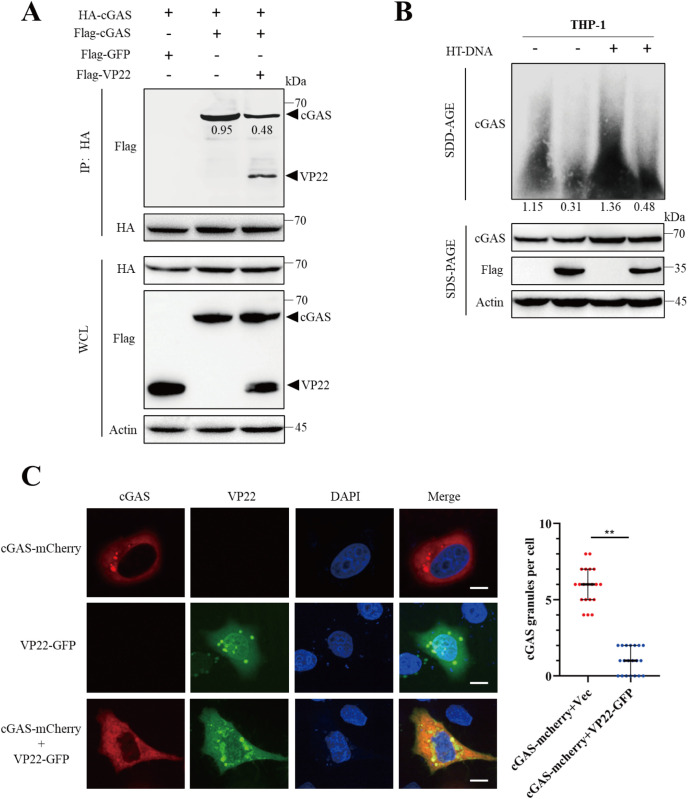
PRV VP22 attenuates cGAS condensation. (A) HEK-293T cells were co-transfected with the indicated expression plasmids for 24 hours. Cell lysates were subjected to immunoprecipitation using anti-HA-conjugated beads, followed by immunoblotting. Both whole-cell lysates (WCLs) and immunoprecipitates were analyzed with antibodies against HA, Flag, and β-actin. (B) THP-1 cells were transfected with the indicated plasmids for 24 hours. Cell lysates were analyzed by semi-denaturing detergent agarose gel electrophoresis (SDD-AGE) or conventional SDS-PAGE, followed by immunoblotting with antibodies against cGAS, Flag, and β-actin. Densitometric quantification of cGAS aggregates is shown below the blot. (C) HEK-293T cells were transfected with cGAS-mcherry along with either Flag-Vector or VP22-GFP for 24 hours. Cells were stained with the live-cell dye Hoechst to label nuclei and were subsequently observed directly under a fluorescence microscope. Representative images were acquired using confocal microscopy. The number of cGAS granules per cell was quantified (right panel), with at least 20 cells analyzed per group. Scale bar: 5 μm. Data are representative of at least three independent experiments with similar results (mean ± SD, n = 20 biological replicates in panel C) ** P < 0.01.

Building on our findings that VP22 binds to cGAS and attenuates its enzymatic activity, we next sought to elucidate the mechanism by which VP22 inhibits cGAS activation. Previous studies have shown that cGAS undergoes DNA-induced conformational changes, forming dimers and higher-order oligomers that are essential for its full enzymatic activation [4]. To investigate whether VP22 affects cGAS self-association, we conducted a co-immunoprecipitation assay. We found that VP22 expression markedly reduced the interaction between HA-cGAS and Flag-cGAS, indicating an inhibitory effect on cGAS oligomerization (Fig 3A). We then examined the formation of higher-order cGAS assemblies and observed that VP22 reduced the presence of cGAS condensates, regardless of HT-DNA stimulation (Fig 3B). To further validate these results, we performed immunofluorescence staining. Consistent with the biochemical results, VP22 overexpression markedly and significantly decreased the number of cGAS cytoplasmic granules (Fig 3C), suggesting impaired formation of functional cGAS condensates. Collectively, these results demonstrate that VP22 disrupts cGAS condensation, thereby impairing its enzymatic activation.

### cGAS is indispensable for regulating PRV ΔVP22 infection

**Fig 4:**
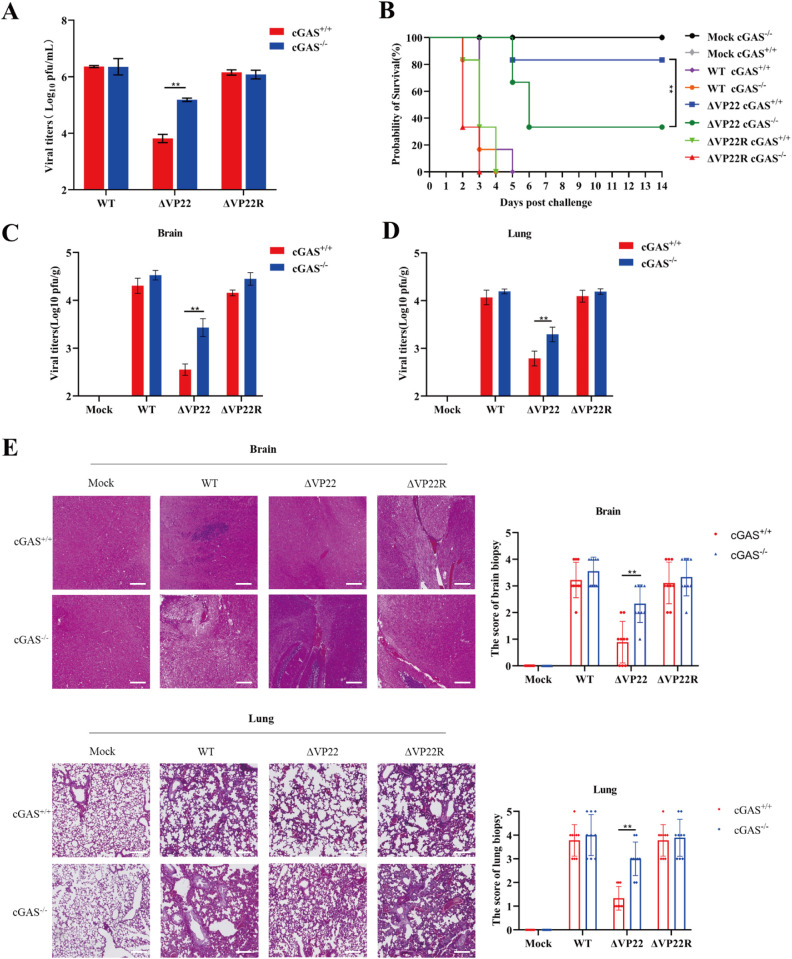
cGAS is essential for controlling PRV ΔVP22 infection. (A) Mouse embryonic fibroblasts (MEFs) from wild-type (WT) and cGAS ⁻ ^/^ ⁻ mice were infected with PRV-WT, ΔVP22, or ΔVP22R at an MOI of 0.01. At 48 hours post-infection (hpi), cells were harvested, and total viral titers were quantified using a plaque assay in Vero cells. (B) Survival of WT and cGAS ⁻ ^/^ ⁻ mice (n = 6 per group) was monitored over time. Mice were mock-infected or intraperitoneally injected with 1 × 10⁴ PFU of PRV-WT, ΔVP22, or ΔVP22R, and survival was recorded as a percentage. (C) WT and cGAS ⁻ ^/^ ⁻ mice (n = 3 per group) were mock-infected or intraperitoneally injected with 1 × 10⁴ PFU of PRV-WT, ΔVP22, or ΔVP22R. At 3 days post-infection (dpi), brains were harvested, and total virus titers were quantified using a plaque assay in Vero cells. (D) Viral titers in lung tissues from the same mice described in panel C. (E) Hematoxylin and eosin (H&E) staining of brain and lung sections from infected mice at 3 dpi (1 × 10⁴ PFU per mouse). Histopathological alterations were further assessed using a scoring system, with three representative sections per mouse evaluated. Scale bars: 100 μm. Data are representative of at least three independent experiments with similar results (mean ± SD, n = 3 biological replicates in panels A, C, D and E). ** P < 0.01.

Next, we investigated the impact of cGAS deficiency on PRV ΔVP22 replication and pathogenesis. Mouse embryonic fibroblasts (MEFs) isolated from wild-type (WT) and cGAS ⁻ ^/^ ⁻ mice were infected with PRV strains (WT, ΔVP22, or ΔVP22R). Plaque assays revealed that ΔVP22 viral titers were significantly higher in cGAS ⁻ ^/^ ⁻ MEFs compared to WT MEFs, while the replication of PRV WT and ΔVP22R was largely unaffected by cGAS deficiency (Fig 4A). To assess the in vivo relevance, ΔVP22 infection resulted in 80% survival in WT mice but only 30% in cGAS ⁻ ^/^ ⁻ mice, underscoring the protective role of cGAS against ΔVP22 infection (Fig 4B). Consistently, PRV plaque counts in the brain and lung tissues of WT mice infected with ΔVP22 were significantly lower than those in cGAS ⁻ / ⁻ mice (Fig 4C and 4D). Similar results were observed for PRV DNA loads (S1 Fig). Moreover, neuropathological lesions in the brain, as well as inflammatory cell infiltration and congestion in the lungs, were noticeably milder in WT mice infected with ΔVP22 compared with those in cGAS ⁻ ^/^ ⁻ mice, with corresponding histopathological scoring showing consistent results (Fig 4E). Taken together, cGAS-dependent cytosolic DNA-sensing pathway plays a critical role in restricting PRV replication, and the depletion of VP22 impairs the ability to counteract the host’s defense mechanisms.

### DDX21 mediates VP22-induced inhibition of cGAS signaling

**Fig 5:**
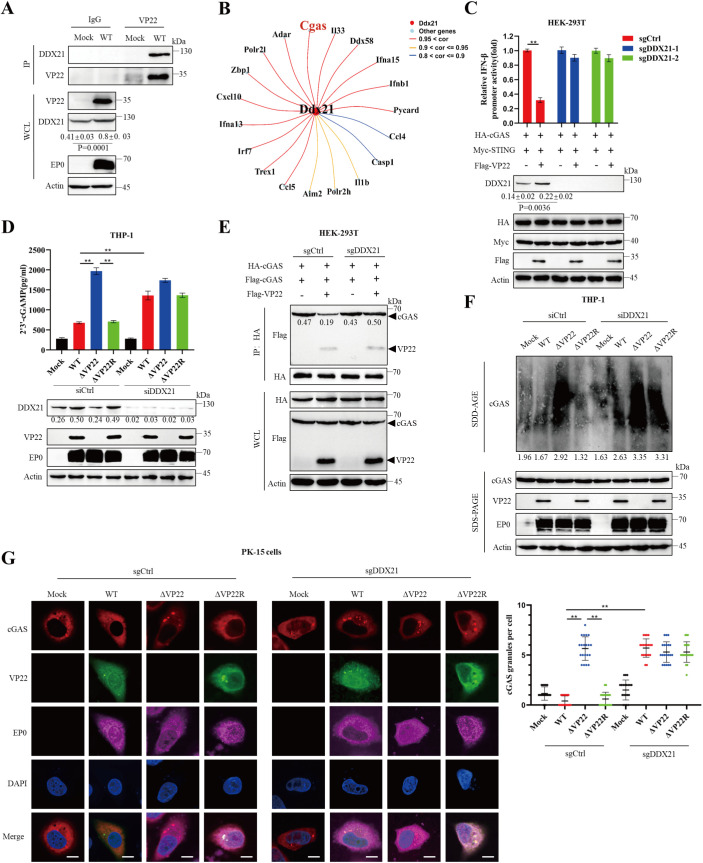
DDX21 mediates VP22-induced suppression of cGAS activity. (A) THP-1 cells seeded in 10-cm dishes were mock-infected or infected with PRV. At 24 hours post-infection (hpi), cell lysates were immunoprecipitated with a mouse anti-VP22 antibody or control IgG, followed by immunoblotting with antibodies against DDX21, VP22, EP0, and β-actin. (B) Correlation analysis between DDX21 and key genes involved in the cytosolic DNA sensing pathway. (C) Wild-type and DDX21-knockout HEK-293T cells were transfected with the indicated plasmids. At 24 hours post-transfection (hpt), cells were lysed for luciferase reporter assays (upper panel) and immunoblotting (lower panels) using antibodies against DDX21, Flag, HA, Myc, and β-actin. (D) THP-1 cells were transfected with siDDX21 or control siRNA (siCtrl). At 48 hpt, cells were infected with PRV-WT, ΔVP22, or ΔVP22R at an MOI of 0.1 for 24 hours. Cytoplasmic cGAMP was extracted and quantified using an ELISA kit (upper panel), and cell lysates were analyzed by immunoblotting (lower panels) with antibodies against DDX21, VP22, EP0 and β-Actin. (E) Wild-type and DDX21-knockout HEK-293T cells were transfected with the indicated plasmids. At 24 hpt, cell lysates were subjected to co-immunoprecipitation (Co-IP) with anti-Flag beads, followed by immunoblotting with antibodies against Flag, HA, β-actin. (F) THP-1 cells were transfected with siDDX21 or siCtrl. At 48 hpt, cells were infected with PRV-WT, ΔVP22, or ΔVP22R. Cell lysates were resolved by semi-denaturing detergent agarose gel electrophoresis (SDD-AGE) or standard SDS-PAGE, followed by immunoblotting using antibodies against cGAS, VP22, EP0 and β-Actin. (G) Wild-type and DDX21-knockout PK15 cells were infected with PRV-WT, ΔVP22, or ΔVP22R for 24 hours. Cells were fixed and stained for cGAS (red), VP22 (green), EP0 (magenta) and nuclei (DAPI, blue), and imaged using confocal microscopy. Scale bars: 10 μm. The number of cGAS granules per cell was quantified (right panel), with at least 20 cells analyzed per group. Data are representative of at least three independent experiments with similar results (mean ± SD; n = 3 biological replicates for panels C and D, n = 20 for panel G). ** P < 0.01.

Given the critical role of host proteins in virus–host interactions, we sought to investigate how VP22 modulates the host innate immune response by identifying cofactors involved in cGAS regulation. To this end, we performed co-immunoprecipitation (Co-IP) coupled with mass spectrometry (MS) analysis. Comparative proteomic profiling revealed host proteins specifically enriched in the VP22 pulldown (S2 Table). Among the top five candidates, only knockout of DDX21 significantly reversed the inhibitory effect of VP22 on interferon promoter activity (S2 Fig). Subsequent co-immunoprecipitation experiments confirmed the interaction between VP22 and DDX21 under both endogenous expression conditions and during viral infection (Figs 5A and S3).

To clarify the functional significance of DDX21 recruitment by VP22, we performed a complementary analysis of transcriptomic profiles from mock- and PRV-infected cells. First, single-gene enrichment analysis based on DDX21 expression revealed strong associations with IFN-α/γ responses and other immune-related pathways (S4A Fig). Second, KEGG enrichment of differentially expressed genes in PRV-infected cells indicated robust activation of the cytosolic DNA sensing pathway (S4B and S4C Fig). Notably, correlation analysis revealed a strong positive association between DDX21 and core gene of the DNA sensing pathway, particularly cGAS (Fig 5B). These findings suggest that DDX21 may mediate VP22-induced suppression of cGAS activity.

To ensure the specificity of the observed phenotype and minimize potential off-target effects, we designed two independent sgRNAs targeting DDX21, both of which had no detectable impact on cell proliferation (S5A Fig). Reporter gene assays confirmed that the inhibitory effect of VP22 on cGAS signaling was abolished in DDX21 knockout cells (Fig 5C). Consistently, VP22-mediated suppression of TBK1 and IRF3 phosphorylation, as well as the mRNA expression and the secretion of IFN-β and other antiviral factors, was also abrogated in DDX21 ⁻ ^/^ ⁻ cells (S5B-S5D Fig). cGAMP, the enzymatic product of cGAS, acts as a second messenger [30], making its quantification essential for evaluating cGAS-mediated immune activation. When THP-1 cells were transfected with siRNA targeting DDX21 or with a control siRNA and subsequently infected with PRV WT, ΔVP22, or ΔVP22R, cGAMP production was markedly increased in DDX21-depleted cells. These results suggest that DDX21 plays a key role in VP22-mediated immune suppression (Figs 5D and S6A).

Furthermore, we investigated whether DDX21 mediates VP22-induced suppression of cGAS condensation and biomolecular droplet formation. Co-immunoprecipitation (Co-IP) assays revealed that DDX21 deficiency enhanced cGAS self-association even in the presence of VP22, suggesting that DDX21 negatively regulates cGAS oligomerization (Fig 5E). Semi-denaturing detergent agarose gel electrophoresis (SDD-AGE) further demonstrated that high molecular weight (HMW) cGAS condensates could still form in DDX21-deficient cells despite VP22 expression (Fig 5F). In addition, cGAS droplet formation was significantly increased in DDX21 ⁻ ^/^ ⁻ cells compared to wild-type cells following infection with either PRV WT or ΔVP22R (Fig 5G), reinforcing the role of DDX21 in modulating cGAS condensation. Notably, it was found that DDX21 deficiency impaired PRV replication, reinforcing its critical role in viral infection (S6B Fig). Collectively, these results suggest that DDX21 is a key host factor mediating VP22-induced inhibition of cGAS activity.

### PRV VP22 stabilizes DDX21 and enhances its interaction with cGAS

**Fig 6:**
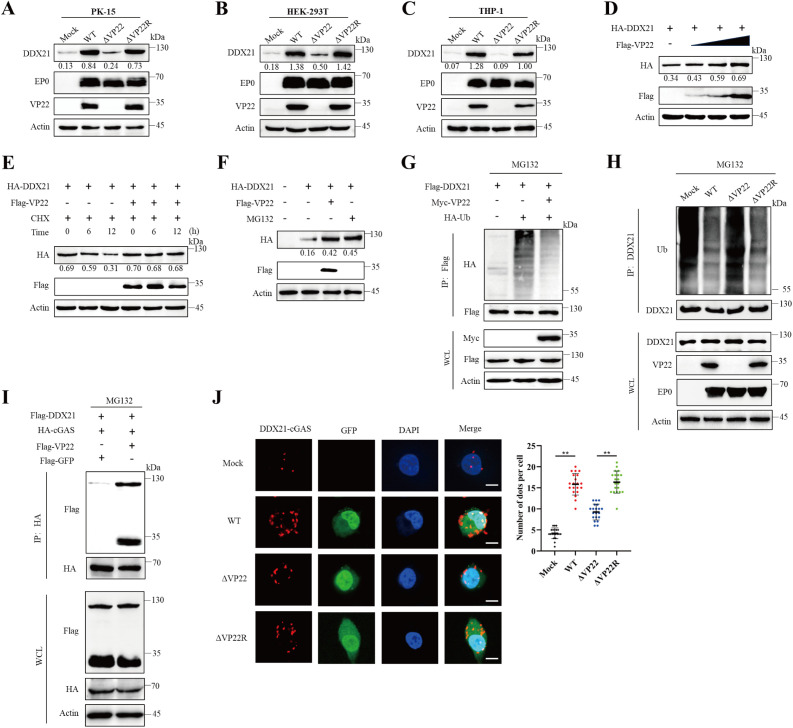
PRV VP22 stabilizes DDX21 and enhances the interaction between DDX21 and cGAS. (A–C) Detection of DDX21 expression following PRV infection in PK-15 (A), HEK-293T (B), and THP-1 (C) cells. Cells were mock-infected or infected with PRV-WT, ΔVP22, or ΔVP22R at an MOI of 0.1 for 24 h, then harvested for western blot analysis using antibodies against DDX21, EP0, VP22, and β-actin. (D) HEK-293T cells were co-transfected with HA-DDX21 and increasing amounts of Flag-VP22. At 24 h post-transfection (hpt), cells were harvested for western blot analysis with antibodies against Flag, HA, and β-actin. (E) HEK-293T cells were co-transfected with HA-DDX21 and Flag-VP22, then treated with cycloheximide (CHX, 100 μg/ml) for the indicated times. Cells were harvested for western blotting with antibodies against Flag, HA, and β-actin. (F) HEK-293T cells were co-transfected with HA-DDX21 and Flag-VP22 for 18 h, followed by treatment with the proteasome inhibitor MG132 (10 μM) for 12 h. Cells were harvested for western blot analysis with antibodies against Flag, HA, and β-actin. (G) Immunoblot analysis of lysates from HEK-293T cells transfected with HA-tagged ubiquitin (HA-Ub) and Flag-DDX21, with or without Myc-VP22, followed by immunoprecipitation (IP) with anti-Flag beads, and immunoblotting with antibodies against Flag, HA, Myc, and β-actin. (H) HEK293T cells were infected with mock, PRV WT, ΔVP22, or VP22R and treated with MG132 (10 μM). Cell lysates were then collected followed by DDX21 immunoprecipitation and immunoblotting with antibodies against Ub, DDX21, VP22, EP0 and β-actin. (I) Co-immunoprecipitation of HA-cGAS with Flag-DDX21 and VP22 in HEK-293T cells. Cells were transfected with the indicated plasmids, followed by treatment with MG132 (10 μM), and then subjected to HA immunoprecipitation and immunoblotting with antibodies against HA, Flag, and β-actin. (J) PK-15 cells were mock-infected or infected with PRV-WT-GFP, ΔVP22-GFP, or ΔVP22R-GFP, then subjected to proximity ligation assay (PLA) using anti-cGAS and anti-DDX21 antibodies. The number of PLA spots was quantified in 20 cells per group. Scale bar: 5 μm. Data represent at least three independent experiments with similar results (mean ± SD, n = 20 biological replicates in J). ** P < 0.01.

As noted above, VP22 overexpression significantly increases DDX21 protein levels (Fig 5A and 5C). To further validate this observation across different cellular contexts, we performed PRV infections in PK-15, THP-1, and HEK-293T cells. In all three cell lines, PRV infection led to upregulated DDX21 protein expression, whereas deletion of VP22 abolished this effect (Fig 6A–C), prompting further investigation into the underlying regulatory mechanism. Interestingly, transcriptomic analysis revealed that PRV infection elevated DDX21 mRNA levels regardless of VP22 expression—a result confirmed by quantitative PCR (S7A and S7B Fig). Moreover, VP22 overexpression alone did not increase DDX21 transcript levels (S7C Fig). These findings suggest that VP22 regulates DDX21 at the post-transcriptional level, enhancing protein abundance without affecting transcription. To elucidate this mechanism, we first confirmed that VP22 increased DDX21 protein levels in a dose-dependent manner (Fig 6D). We then treated cells with the protein synthesis inhibitor cycloheximide (CHX) and observed that VP22 prolonged DDX21 protein stability (Fig 6E). Furthermore, treatment with the proteasome inhibitor MG132 restored DDX21 levels similarly to VP22 overexpression (Fig 6F), suggesting that DDX21 undergoes proteasome-mediated degradation, which is counteracted by VP22. To explore this further, we assessed DDX21 ubiquitination following VP22 treatment. Notably, VP22 overexpression substantially reduced DDX21 ubiquitination compared with the control (Fig 6G). Consistently, under endogenous conditions during viral infection, the VP22-deficient group exhibited significantly higher levels of DDX21 ubiquitination than the WT infection group (Fig 6H).

Given the role of DDX21 in mediating VP22-induced suppression of cGAS activation, we next asked whether DDX21 interacts with cGAS. Co-immunoprecipitation confirmed the interaction between DDX21 and cGAS (S8A and 8B Fig). Building on this, we examined whether VP22 enhances the interaction between DDX21 and cGAS. Co-immunoprecipitation (co-IP) experiments showed that VP22 significantly enhanced the interaction between DDX21 and cGAS (Fig 6I), and the addition of MG132 excluded the possibility that this enhanced interaction was due to changes in DDX21 expression levels. To directly visualize this interaction in situ, we employed a proximity ligation assay (PLA), a technique capable of detecting protein interactions within 40 nm. Consistent with our biochemical data, PRV VP22 markedly increased the number of PLA signals, indicating a robust enhancement of the DDX21–cGAS interaction (Fig 6J). To rule out the possibility that the reduced interaction observed in the absence of VP22 was caused by decreased viral DNA levels and impaired cGAS activation, we measured viral DNA after infection and found no significant difference between WT and VP22-deficient viruses (S8C Fig). Moreover, DNase treatment markedly diminished the DDX21-cGAS interaction, indicating that viral DNA contributes to this process (S8D Fig). Together, these findings demonstrate that VP22 stabilizes DDX21 protein levels and promotes its interaction with cGAS, thereby contributing to the suppression of cGAS-mediated antiviral responses.

### The cytoplasmic localization of DDX21 mediates VP22-induced cGAS inhibition

**Fig 7:**
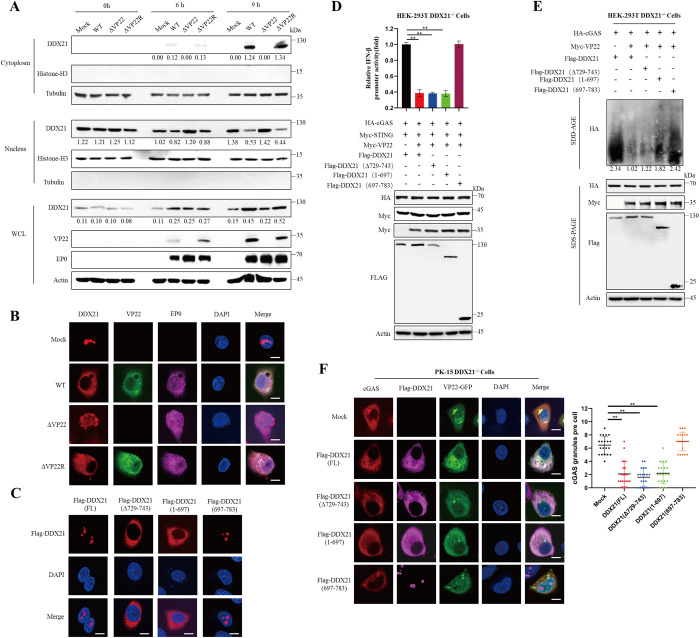
The cytoplasmic localization domain of DDX21 is critical for VP22-induced suppression of cGAS signaling. (A) PK-15 cells were mock-infected or infected with PRV-WT, ΔVP22, or ΔVP22R at an MOI of 1, and cells were harvested at 0-, 6-, and 9-hours post-infection. Cytoplasmic and nuclear fractions were isolated and analyzed by immunoblotting with antibodies against Histone H3, Tubulin, DDX21, VP22, EP0, and β-actin. Histone H3 and Tubulin were used as markers for nuclear and cytoplasmic fractions, respectively. (B) PK-15 cells were mock-infected or infected with PRV-WT, ΔVP22, or ΔVP22R at an MOI of 1 for 9 hours. Cells were fixed and subjected to immunofluorescence staining to detect endogenous DDX21 (green), VP22 (red), and nuclei (DAPI, blue). Representative images are shown. Scale bars: 5 μm. (C) HEK-293T cells were transfected with full-length DDX21 or its truncated mutants (DDX21(Δ729-743), 1–697 and 697–783). After 24 hours, cells were processed as in (B). Representative images are shown. Scale bars: 5 μm. (D) Wild-type and DDX21-knockout HEK-293T cells were co-transfected with Flag-DDX21 (full-length or truncated mutants) and the indicated expression plasmids for 24 hours. IFN-β promoter-driven luciferase activity was measured (top panel). Parallel immunoblotting was performed using antibodies against HA, Myc, Flag, and β-actin (bottom panel). (E) Wild-type and DDX21-knockout HEK-293T cells were co-transfected as in (D). Semi-denaturing detergent agarose gel electrophoresis (SDD-AGE) was performed to examine cGAS protein aggregation, followed by immunoblotting with antibodies against Flag, HA, and β-actin. (F) Wild-type and DDX21-knockout PK-15 cells treated as in (D) were subjected to immunofluorescence staining to detect cGAS (red), DDX21 (magenta) and VP22 (green). Confocal microscopy quantified the number of cGAS puncta per cell, analyzing at least 20 cells per group. Scale bars: 5 μm. Data represent at least three independent experiments with similar results (mean ± SD; n = 3 biological replicates in D, n = 20 biological replicates in F). ** P < 0.01.

The immunoregulatory function of DDX21 is closely tied to its subcellular localization. Under basal conditions, DDX21 is predominantly nuclear, but it can translocate to the cytoplasm in response to viral infection or cellular stress, where it actively participates in innate immune signaling [17,19,31,32]. Based on these observations, we hypothesized that VP22 manipulates DDX21 localization to disrupt innate immune responses. To test this, we performed nuclear–cytoplasmic fractionation followed by Western blotting. The results showed that in cells infected with WT or ΔVP22R virus, DDX21 was redistributed from the nucleus to the cytoplasm, whereas in ΔVP22-infected cells, it largely remained in the nucleus (Fig 7A). Consistently, immunofluorescence assays in PK-15 cells demonstrated that DDX21 predominantly localized to the cytoplasm during WT and ΔVP22R infection but was retained in the nucleus during ΔVP22 infection (Fig 7B).

As previous studies have demonstrated that the C-terminal region of DDX21 plays a crucial role in regulating its nuclear localization signal [33–35], we aimed to identify the specific region of DDX21 responsible for its nuclear retention by constructing truncation mutants. Our results showed that DDX21(Δ729–743), which lacks key amino acids required for nuclear translocation, and the truncation mutant DDX21(1–697), which lacks the C-terminal amino acids 697–783, both exhibited markedly reduced nuclear localization (Fig 7C). To assess whether this region is also required for DDX21’s immunomodulatory function, we conducted dual-luciferase reporter assays in DDX21-knockout cells reconstituted with either full-length Flag-DDX21, DDX21(Δ729–743), the N-terminal truncation (1–697), or the C-terminal fragment (697–783). The results showed that DDX21(FL), DDX21(Δ729–743), and the N-terminal truncation (1–697) suppressed cGAS-induced IFN-β promoter activity in the presence of VP22 (Fig 7D).

We further investigated which region of DDX21 mediates VP22-induced inhibition of cGAS condensation by examining cGAS oligomerization and droplet formation. Notably, only full-length DDX21, DDX21 (Δ729–743) and the N-terminal truncation (1–697) supported cGAS aggregation (Fig 7E). Consistently, confocal microscopy showed that VP22 suppressed cGAS condensate formation only in cells reconstituted with full-length DDX21, DDX21 (Δ729–743) and the N-terminal truncation (1–697), whereas cells expressing the C-terminal truncation (697–783) failed to restore this suppression (Fig 7F). Taken together, these results suggest that PRV VP22 promotes and relies on DDX21 redistribution to the cytoplasm, thereby facilitating viral evasion of cGAS-mediated innate immunity.

## Discussion

Although extensive research has shed light on how viral proteins antagonize host innate immune pathways, most studies focused on the direct inhibition of key immune sensors or signaling intermediates [36]. Many DNA viruses, such as HSV-1 and vaccinia virus (VACV), encode proteins that directly target cGAS or STING through mechanisms such as degradation, ubiquitination, or competitive binding [37–39]. For example, HSV-1 UL37 binds to and inhibits the enzymatic activity of cGAS [40,41], while KSHV ORF52 sequesters cytosolic DNA to prevent its detection by cGAS [42]. Similarly, the VACV E3 protein masks immunostimulatory DNA, thereby impairing cGAS activation [43]. However, comparatively little is known about how viruses hijack host regulatory proteins—particularly those that modulate the biophysical states of immune effectors—to indirectly suppress immune activation [44,45]. This presents a critical conceptual and technical gap in our understanding of immune evasion: specifically, how viruses manipulate host cofactors to reprogram the phase behavior, spatial organization, or functional activation of pattern recognition receptors such as cGAS [37,46–48]. Our study fills this gap by identifying a novel mechanism through which pseudorabies virus (PRV) evades immune detection. We showed that VP22 modulates the physical state and interaction dynamics of cGAS by regulating the nucleocytoplasmic distribution of the host RNA helicase DDX21, eventually suppressing cGAS-mediated innate immune activation.

cGAS plays a central role in detecting viral DNA and initiating type I interferon responses, functioning as a crucial first line of defense against DNA virus infections. Due to its importance, cGAS is frequently targeted by viral immune evasion strategies. Our results extend previous observations from HSV to PRV, showing that VP22 similarly modulates this pathway and further highlighting cGAS as a key viral vulnerability. Notably, we previously demonstrated that VP22 also inhibits ZBP1-mediated activation of the NLRP3 inflammasome and necroptotic signaling [29]. ZBP1 is another cytosolic DNA sensor that recognizes Z-form nucleic acids and induces inflammatory and cell death responses via RIPK3 [49,50]. Although cGAS and ZBP1 engage distinct downstream effectors—STING and RIPK3, respectively—they both function as early sensors of aberrant cytosolic DNA during viral infection [51]. Therefore, determining whether VP22 targets conserved domains or structural motifs shared by cGAS and ZBP1 is critical to advancing our understanding of PRV and broader herpesvirus immune evasion strategies. Alternatively, VP22 may interfere with a common regulatory node to simultaneously suppress both DNA sensing pathways.

Our findings identify DDX21 as a critical host factor exploited by PRV to suppress cGAS-mediated innate immune signaling. The VP22-induced translocation of DDX21 from the nucleus to the cytoplasm appears essential for inhibiting cGAS condensation and enzymatic activation. Notably, while PRV infection significantly upregulates DDX21 mRNA levels in a VP22-independent manner, the increase in DDX21 protein expression requires the presence of VP22. This suggests that further investigation is needed to elucidate the precise mechanisms by which PRV regulates DDX21 transcription. Intriguingly, our results indicate that VP22 enhances DDX21 stability by inhibiting its ubiquitination, thereby facilitating immune evasion. Whether VP22 achieves this by modulating specific deubiquitinating enzymes remains an open question. Addressing these issues will be key to advancing our understanding of how PRV manipulates host post-translational modification systems to evade antiviral defenses.

In summary, our findings reveal a previously unrecognized immune evasion mechanism by which the PRV tegument protein VP22 co-opts the host RNA helicase DDX21 to impair cGAS activation. This study expands the current paradigm of viral immunomodulation beyond direct antagonism, highlighting cofactor-mediated regulation of cGAS condensation as a novel frontier in host–pathogen interactions.

## Materials and methods

### Ethics statement

All mouse experiments were performed according to the National Guidelines for Housing and Care of Laboratory Animals (China) and Institutional Animal Care and Ethics Committee of Nanjing Agricultural University (permit no. IACECNAU20210602). All mice were housed in the animal facility of Nanjing Agricultural University (Nanjing, Jiangsu, China).

### Cells, viruses and plasmids

Porcine kidney (PK-15; CCL-33), human embryonic kidney (HEK-293T; CRL-11268), Vero (CCL-81), and human acute monocytic leukemia (THP-1; TIB-202) cell lines were obtained from the American Type Culture Collection (ATCC). DDX21-knockout cell lines were generated using a lentiviral sgRNA system, followed by selection with puromycin at 2 µg/mL (Selleck, #S9631). PK-15, HEK-293T, and Vero cells were cultured in Dulbecco’s Modified Eagle Medium (DMEM; Gibco, #11995065) supplemented with 10% fetal bovine serum (FBS; Gibco, #10099141). THP-1 cells were maintained in RPMI 1640 medium (Gibco, #12633020) supplemented with 15% FBS. For differentiation into adherent macrophage-like cells, THP-1 cells were treated with 80 nM phorbol 12-myristate 13-acetate (PMA; Sigma-Aldrich, FMS-FZ207) for 24 hours. All cells were incubated at 37°C in a humidified atmosphere containing 5% CO₂.

The pseudorabies virus (PRV) wild-type (WT) strain SXRH-10/2023 (GenBank: PP097194.1) was used in this study [52]. Recombinant viruses lacking the VP22 gene (ΔVP22) and the corresponding revertant (ΔVP22R) were generated as previously described [29], consistent with earlier approach [23]. Fusion viruses, including PRV-WT-GFP, PRV-ΔVP22-GFP, and PRV-ΔVP22R-GFP, were constructed through homologous recombination. GFP was fused to the PRV VP5 protein, and recombinant viruses were isolated through multiple rounds of plaque purification. The resulting clones were verified by sequencing, and the construction method has been described previously [53]. Virus stocks were prepared, and infectivity titrations were performed in Vero cells.

Expression plasmids including Flag-VP22, Flag-cGAS, HA-cGAS, Myc-STING, Flag-GFP, HA-GFP, HA-Ub, and the lentiviral packaging plasmids pMD2.G and psPAX2 were described previously [23]. IFN-β-Luc and pRL-TK were described elsewhere [54]. DDX21 was obtained by inserting human DDX21 cDNA between the *BamH*I and *Xho*I sites of pCMV-N-HA and pCDH Flag. Flag-DDX21 was used as a PCR template to generate the truncation mutants, also inserting into the *BamH*I and *Xho*I sites of pCDH-Flag, specifically Flag-DDX21(Δ729–743), Flag-DDX21 (1–697 aa) and Flag-DDX21 (697–783 aa). All the constructs were confirmed by Sanger sequencing (Sangon Biotech). The primers used for plasmid construction are listed in S1 Table.

### Antibodies and chemical reagents

The antibodies used in this study were as follows: anti-FLAG (HRP-conjugated, M2; Sigma-Aldrich, #A8592), anti-HA (HRP-conjugated, 6E2; Cell Signaling Technology, #2999), anti-Myc (HRP-conjugated, 9B11; Cell Signaling Technology, #2040), anti-β-actin (AC-15; Sigma-Aldrich, #A5441), anti-cGAS (human-specific, E5V3W; Cell Signaling Technology, #79978), anti-cGAS (mouse-specific, D3O8O; Cell Signaling Technology, #31659), anti-cGAS (monoclonal; Proteintech, #68640–1-Ig), anti-phospho-TBK1 (Ser172, D52C2; Cell Signaling Technology, #5483), anti-TBK1 (D1B4; Cell Signaling Technology, #3504), anti-phospho-IRF3 (Ser396, 4D4G; Cell Signaling Technology, #4947), anti-IRF3 (D83B9; Cell Signaling Technology, #4302S), anti-DDX21 (human-specific, EPR14495; Abcam, #ab182156), anti-DDX21 (mouse-specific; AFW7034), anti-Histone H3 (Proteintech, #10445–1-AP), and anti-α-Tubulin (Proteintech, #80762–1-RR). Secondary antibodies included mouse anti-rabbit IgG-HRP (Santa Cruz Biotechnology, #sc-2357) and goat anti-mouse IgG-HRP (Santa Cruz Biotechnology, #sc-2005). Anti-VP22 and anti-EP0 antibodies were generated in-house and used in this study [29].

The following chemical reagents were used: herring testes DNA (HT-DNA; Sigma-Aldrich, #D6898), MG-132 (Selleck, #S2619), cycloheximide (CHX; Selleck, #S7418), and phorbol 12-myristate 13-acetate (PMA; Sigma-Aldrich, #FMS-FZ207). **Luciferase reporter analysis and transfection.**

HEK-293T cells were transfected using Lipofectamine 3000 (Thermo Fisher Scientific, #L3000015) according to the manufacturer’s instructions. A firefly luciferase reporter plasmid encoding IFN-β-Luc and a Renilla luciferase control plasmid (pRL-TK) were co-transfected along with the indicated expression plasmids. An empty vector was included to ensure equal total DNA amounts across all transfections. Cells were lysed for luciferase assays, and firefly luciferase activity was normalized to Renilla luciferase activity.

### Real-time qPCR

Total RNA was extracted from cells using the RNeasy Plus Mini Kit (Qiagen, #74104) according to the manufacturer’s instructions. Approximately 200 ng of RNA was used for cDNA synthesis with the HiScript II Q RT SuperMix for qPCR (Vazyme, #R223-01). Quantitative PCR was performed using an Applied Biosystems ABI Prism 7900HT instrument with AceQ qPCR SYBR Green Master Mix (Vazyme, #Q111-02). For transcriptomic analysis, gene expression levels were normalized to the endogenous control 18S rRNA. Relative gene expression was calculated as previously described [55]. Primer sequences for human *IFN-β*, *IFIT1*, *IFIT2*, *ISG15*, and *18S rRNA* are listed in S1 Table.

### Plasmids and siRNA transfections

Plasmid transfections in HEK-293T cells were performed using Lipofectamine 3000 (ThermoFisher Scientific, L3000015), while transfections in PK-15 cells were carried out using Lipofectamine LTX & Plus Reagent (ThermoFisher Scientific, #15338100). siRNA transfections were conducted using Lipofectamine RNAiMAX (ThermoFisher Scientific, #13778075). All procedures were performed according to the manufacturers’ instructions. The siRNA sequences are listed in S1Table.

### Generation of knockout cell lines by CRISPR-Cas9 system

The LentiCRISPRv2-puro plasmid containing a DDX21-targeting sgRNA and the corresponding non-targeting sgRNA control plasmid (negative control) were constructed using the BsmBI digestion method. Briefly, the LentiCRISPRv2 vector was digested with BsmBI to generate a linearized backbone. Synthesized targeting or non-targeting sgRNA oligonucleotides were then annealed and ligated into the digested vector using T4 DNA ligase. The recombinant plasmids were obtained through transformation and selection in E. coli. Pooled knockout PK-15 and HEK-293T cells were generated by lentiviral transduction. For this, the LentiCRISPRv2 vector expressing Cas9 and sgRNAs targeting DDX21, RCC6, SSBP1, PGAM5, or KEAP1, together with the packaging plasmids (pMD2.G and psPAX2), were co-transfected into HEK-293T cells. Lentivirus-containing supernatants were collected 48 hours post-transfection (hpt) and used to infect target cells. At 16 hours post-infection (hpi), the medium was replaced with fresh culture medium. At 3 days post-infection (dpi), cells were selected with 2 μg/mL puromycin (Santa Cruz Biotechnology). Single-cell clones of knockout HEK-293T and PK-15 cells were isolated by serial dilution into 96-well plates and screened for gene knockout by Western blotting and Sanger sequencing. All experiments were performed within two weeks after lentiviral transduction.

### VSV-GFP infection inhibition assay

GFP fluorescence from VSV-GFP was used as a readout to evaluate antiviral cytokine secretion. Supernatants collected from the dual-luciferase assay were added to fresh, confluent HEK-293T cells and incubated for 24 hours. The cells were then infected with VSV-GFP at a multiplicity of infection (MOI) of 0.01. At 24 hours post-infection (hpi), VSV-GFP replication was assessed by monitoring GFP expression using fluorescence microscopy (Zeiss, Axio Vert.A1).

### Viral infection

Cells were infected with viruses at the indicated multiplicity of infection (MOI). After 2 hours of adsorption, the monolayers were overlaid with DMEM supplemented with 1% FBS and incubated at 37°C. For viral titer determination, samples were collected at 48 hpi, and viruses were released by three freeze–thaw cycles. Viral titers were determined by plaque assay on Vero cells, as previously described [54]. For western blot assay, the cell lysates were harvested at indicated timepoints for determination.

### Mass spectrometry analysis

HEK-293T cells were transfected with either Flag-GFP or Flag-VP22. At 48 hpt, cells were harvested and lysed in NP-40 lysis buffer (50 mM Tris-HCl, pH 7.4; 1% NP-40 [Beyotime, P0013F]; 150 mM NaCl; 1 mM EDTA; and 1:400 protease inhibitor cocktail [Beyotime, ST506]). After centrifugation at 12,000 × g for 10 minutes, the supernatants were collected and incubated with 50 μL of anti-Flag M2 magnetic beads for 4 hours at 4°C. The immunoprecipitates were extensively washed with lysis buffer and subjected to liquid chromatography–tandem mass spectrometry (LC-MS/MS) using a Q Exactive mass spectrometer (ThermoFisher Scientific, 0726055) coupled with an Easy-nLC 1000 system (ThermoFisher Scientific, LC120). MS data were analyzed using the MASCOT software. Experiments were performed at the Beijing Genomics Institute (BGI).

### Confocal fluorescence microscopy

Cells seeded onto confocal dishes were transfected with the indicated plasmids. At 24 hpt, cells were fixed with 4% paraformaldehyde (Beyotime, P0099) for 20 minutes at room temperature, followed by three washes with phosphate-buffered saline (PBS; Beyotime, C0221A). Cells were then permeabilized with 0.1% Triton X-100 (Beyotime, P0096) in PBS for 10 minutes and blocked with 5% bovine serum albumin (BSA; Beyotime, ST2254) for 1 hour. Subsequently, cells were incubated overnight at 4°C with primary antibodies. After washing with PBS, cells were incubated with a fluorescein-conjugated secondary antibody (Proteintech, SA00013) for 1 hour. Finally, cells were washed with PBS and stained with 4’,6-diamidino-2-phenylindole (DAPI; Sigma-Aldrich, D9542). Stained cells were imaged using a Nikon A1 confocal microscope (Nikon) equipped with a 60 × oil immersion objective.

### Western blot

Proteins from cell lysates were separated by electrophoresis on 8%, 10%, or 12% Bis-Tris SDS-PAGE gels (pH 6.4). The proteins were then transferred onto polyvinylidene difluoride (PVDF) membranes and blocked for 2 hours in 3% (w/v) BSA prepared in phosphate-buffered saline with Tween-20 (PBST; Beyotime, ST825) or Tris-buffered saline with Tween-20 (TBST) for phosphorylated protein detection. The membranes were incubated overnight at 4°C with the indicated primary antibodies. After washing with PBST or TBST, membranes were incubated with HRP-conjugated secondary antibodies diluted in 3% (w/v) BSA in PBST or TBST for 1 hour at room temperature. Signals were detected using Chemistar High-Sig ECL western blot substrate (Tanon, 4600) and imaged on a Tanon 5200 system (Tanon).

### Immunoprecipitation

For immunoprecipitation, cells were harvested and lysed in NP-40 buffer (50 mM Tris-HCl, pH 7.4; 1% NP-40; 150 mM NaCl; 1 mM EDTA; protease inhibitor cocktail at 1:400). After centrifugation, the supernatants were collected and incubated with 50 μL of anti-Flag M2 magnetic beads (Sigma-Aldrich, M8823), anti-Myc magnetic beads (Santa Cruz Biotechnology, sc-500,772), or anti-HA magnetic beads (Cell Signaling Technology, 11846) at 4°C for 6 hours. The beads were then washed three times with wash buffer (50 mM Tris-HCl, pH 7.4; 150 mM NaCl; 5 mM EDTA; 0.1% Triton X-100; protease inhibitor cocktail) before being collected. Precipitated proteins were analyzed by western blot using the indicated antibodies.

### cGAS enzyme activity assays and cGAMP quantification

For measurement of cGAMP levels, a 2’-3’ cGAMP ELISA kit (Cayman Chemical, Cat# 501700) was used. Briefly, cells were transfected with HT-DNA using Lipofectamine 3000 or infected with PRV. At the indicated time points, cells were harvested and lysed in 100 µL of hypotonic buffer (10 mM Tris-HCl, pH 7.5, 5 mM KCl, and 3 mM MgCl₂). The lysates were incubated on ice for 10 minutes, followed by centrifugation to remove denatured proteins. The resulting supernatants were used to measure cGAMP levels. The ELISA was performed according to the manufacturer’s protocol.

### SDD-AGE assay

For detection of cGAS aggregates, cells were transfected or infected as indicated and then lysed in lysis buffer (0.5% Triton X-100, 50 mM Tris-HCl, 150 mM NaCl, 10% glycerol). The supernatants were mixed with 1 × SDD loading buffer (0.5 × TBE, 10% glycerol, 2% SDS, 0.0025% bromophenol blue) and loaded onto a vertical 1.5% agarose gel (1 × TBE, 1.5% agarose). Electrophoresis was carried out in 1 × TBE running buffer containing 0.1% SDS at 4°C for 35 minutes under a constant voltage of 100 V, followed by immunoblotting [56]. Reactions were stopped by adding loading buffer without 2-mercaptoethanol, and the samples were resolved by SDD-AGE as described above.

### Proximity ligation assay (PLA)

PK-15 cells were either mock-infected or infected with PRV-WT-GFP, PRV-ΔVP22-GFP, or PRV-ΔVP22R-GFP (MOI = 1) for 9 hours. PLA was performed using the Duolink In Situ Detection Reagents Green (Sigma-Aldrich, DUO92014). According to the manufacturer’s instructions, cells were sequentially fixed, recovered, and permeabilized. They were then blocked with Duolink blocking buffer at 37 °C for 1 hour, followed by incubation with rabbit anti-cGAS antibody and mouse anti-DDX21 antibody at 37 °C for 2 hours. Next, cells were treated with pre-diluted anti-rabbit and anti-mouse minus probes at 37 °C for 1 hour. Finally, cells were incubated with 1 × ligase for 30 minutes and 1 × polymerase for 100 minutes, then mounted on slides with Duolink In Situ Mounting Medium containing DAPI.

### RNA-seq analysis

RNA-seq data used in this study were obtained from our previous research [29]. To investigate the biological pathways associated with DDX21, we first performed differential expression analysis to identify differentially expressed genes (DEGs) between Mock and WT. Since DDX21 itself was among the DEGs, we next assessed its correlation with all other DEGs across samples and ranked the genes by correlation coefficient, from highest positive to highest negative. This ranked list was then analyzed by preranked Gene Set Enrichment Analysis (GSEA) using curated reference sets from the Molecular Signatures Database [57]. Interferon-related signaling pathways emerged as the most significantly enriched, with the cytolytic DNA pathway also ranking among the top five. To further investigate, we constructed a correlation network linking DDX21 to hub genes within the cytolytic DNA pathway.

### Mice

cGAS^−/−^ mice and wild-type (WT) mice were housed in specific pathogen-free barrier facilities at Nanjing Agricultural University [23]. Mouse embryonic fibroblasts (MEFs) were isolated from 15-day post-coitum embryos collected from euthanized pregnant mice. The isolated cells were plated in culture flasks and maintained in DMEM supplemented with 10% FBS.

### PRV infection in mice and tissue collection

Mice were acclimated for one week before infection challenge experiments. cGAS^−/−^ and WT mice (6–8 weeks old) were housed separately and divided into eight groups (n = 6 per group): (1) mock-infected, (2) PRV-WT-infected, (3) ΔVP22-infected, and (4) ΔVP22R-infected. Mice were intraperitoneally injected with 0.1 mL of a 1 × 10⁴ PFU PRV solution, while mock controls received an equal volume of sterile saline [58]. Clinical symptoms and mortality were monitored daily, and survival rates were calculated using the Reed-Muench method. After 14 days, mice were euthanized using a gradual-fill CO₂ system (20% chamber volume/min), in accordance with animal welfare guidelines, ensuring a humane loss of consciousness. Brain and lung tissues were collected and stored at −80 °C for further analysis.

### Measurement of PRV Viral and DNA loads in mouse tissues

Brain and lung tissues from six mice per group were dissected. Each 100 mg tissue sample was homogenized in 1 mL PBS, and homogenates were briefly centrifuged to collect the supernatant. Viral titers were determined by plaque assay on Vero cells, as previously described [54]. Viral DNA was extracted from supernatants and quantified by qRT-PCR using a standard curve to calculate viral genome copies. Absolute quantification of the PRV gB gene was performed with the FastPure Viral DNA/RNA Mini Kit (Vazyme, Nanjing, China), following established protocols [59]. Primer sequences are listed in S1 Table.

### Hematoxylin-eosin staining and histopathological scoring

Tissues were fixed in 4% paraformaldehyde for >24 h, dehydrated through graded ethanol solutions, and incubated in 1:1 xylene/ethanol for 5 min, followed by two sequential 10 min incubations in pure xylene. Specimens were embedded in paraffin, sectioned, and stained with hematoxylin and eosin (H&E) according to standard protocols. Slides were examined by light microscopy. Lung tissues were assessed for inflammation in airway, vascular, and parenchymal regions, while brain tissues were evaluated for inflammatory infiltrates, necrosis, and tissue damage. Severity was scored on a scale of 0–5: 0 (none), 1 (minimal), 2 (mild/limited), 3 (moderate), 4 (severe/widespread), and 5 (extensive/most prominent) [60].

### Statistical analysis

All data were analyzed using GraphPad Prism 7.0 software (GraphPad Software, Inc.) and are presented as means ± SD. Student’s t-test was used to compare differences between two groups for normally distributed data. A one-way ANOVA with Dunnett’s test was used to compare differences among three groups. A two-way ANOVA with Tukey’s or Sidak’s multiple-comparisons test was used to evaluate experiments involving multiple groups. The P values were calculated from three biological replicates unless otherwise indicated in the figure legends. Data were reproduced in independent experiments as indicated in the legends.

## Supporting information

S1 Fig PRV DNA loads in brain and lung tissues. (A)WT and cGAS ⁻ ^/^ ⁻ mice (n = 3 per group) were mock-infected or intraperitoneally injected with 1 × 10⁴ PFU of PRV-WT, ΔVP22, or ΔVP22R. At 3 days post-infection (dpi), brains were harvested, and total virus DNA loads were measured by RT PCR. (B) virus DNA loads in lung tissues from the same mice described in panel A. Data represent at least three independent experiments with similar results (mean ± SD, n = 3 biological replicates). ** P < 0.01.
(TIF)

S2 Fig DDX21 is involved in PRV VP22-mediated suppression of cGAS. (A) Mass spectrometry (MS) analysis of proteins co-immunoprecipitated with Flag-VP22 from HEK-293T cells. The top five candidate VP22-interacting proteins are listed, ranked by abundance. DDX21 (highlighted in red), a nucleolar RNA helicase, was identified as a top interactor (ranked third). (B) HEK-293T cells with indicated gene knockouts were seeded in 24-well plates and co-transfected with IFN-β-Luc, pRL-TK, HA-cGAS, or Myc-STING, together with Flag-VP22. At 24 h post-transfection (hpt), cells were lysed for luciferase reporter assays. Data represent at least three independent experiments with similar results (mean ± SD, n = 3 biological replicates). ** P < 0.01.
(TIF)

S3 Fig PRV VP22 interacts with DDX21. (A) HEK-293T cells were co-transfected with Flag-VP22 and either HA-GFP or HA-DDX21. At 24 h post-transfection (hpt), cell lysates were immunoprecipitated (IP) with anti-Flag beads, and both whole-cell lysates (WCLs) and precipitates were analyzed by immunoblotting with anti-HA, anti-Flag, and anti-β-actin antibodies. (B) HEK-293T cells were co-transfected with HA-DDX21 and either Flag-GFP or Flag-VP22. At 24 hpt, lysates were immunoprecipitated with anti-HA beads and analyzed as in panel A. Data are representative of three independent experiments.
(TIF)

S4 Fig GSEA reveals DDX21-associated functions during PRV infection. (A) Single-gene GSEA of DDX21-associated genes indicates activation of the Interferon Gamma and Interferon Alpha response pathways. (B) KEGG pathway GSEA based on RNA-seq data from PRV-infected versus mock-treated groups highlights enrichment of the cytosolic DNA-sensing pathway. Bar heights represent NES values, and colors indicate FDR values. (C) GSEA of differentially expressed genes between PRV-infected and mock-treated groups shows significant enrichment of the cytosolic DNA-sensing pathway.
(TIF)

S5 Fig PRV VP22 inhibits the cGAS–STING–mediated type I interferon signaling pathway in a DDX21-dependent manner. (A) HEK-293T cells were transduced with sgRNAs targeting DDX21 (sg-1, sg-2) or a non-targeting control (sgCtrl). Cell numbers were then counted at 0, 12-, 24-, 36-, and 48-hours post-inoculation. (B) Wild-type and DDX21-knockout HEK-293T cells were transfected with HA-cGAS and Myc-STING along with either Flag-vector or Flag-VP22. After 24 hours, total RNA was extracted and the mRNA levels of *IFN-β*, *ISG15*, *IFIT1*, and *IFIT2* were measured by RT-qPCR. Gene expression was normalized to *18S rRNA*. (C) Wild-type and DDX21-knockout HEK-293T cells were transfected with HA-cGAS, Myc-STING, and Flag-VP22 for 24 hours. Cell lysates were subjected to western blot analysis using antibodies against Flag, IRF3, phosphorylated IRF3 (p-IRF3), TBK1, phosphorylated TBK1 (p-TBK1), and β-actin. (D) Immunofluorescence analysis of GFP signal in wild-type and DDX21-knockout HEK-293T cells treated as in panel B. Supernatants were transferred to fresh HEK-293T cells, which were then infected with VSV-GFP at an MOI of 0.01. GFP fluorescence was detected 24 hours post-infection. Scale bars: 100 μm. Data are representative of at least three independent experiments with similar results (mean ± SD, n = 3 biological replicates in A and B). ** P < 0.01.
(TIF)

S6 Fig DDX21 promotes PRV replication. (A) THP-1 cells were transfected with siRNAs targeting DDX21 (si#1) or a non-targeting control (siNC). After 48 hours, cell viability was assessed using a CCK-8 assay (top), and knockdown efficiency was evaluated by immunoblotting (bottom) with antibodies against DDX21 and β-actin. (B) Wild-type and DDX21 ⁻ ^/^ ⁻ PK-15 and HEK-293T cells were infected with PRV at an MOI of 0.01 for 48 hours. Separately, THP-1 cells transfected with siDDX21 or siCtrl for 48 hours were also infected with PRV at an MOI of 0.01 for 48 hours. Viral titers in the supernatants were measured by plaque assay. Data are presented as mean ± SD of n = 3 biological replicates. ** P < 0.01.
(TIF)

S7 Fig PRV infection increases DDX21 mRNA expression. (A) Volcano plots showing differentially expressed genes comparing mock-infected vs. PRV WT (left) and mock-infected vs. PRV ΔVP22 (middle). The heatmap (right) displays the relative expression levels of DDX21 among mock, WT, and ΔVP22 groups. (B) Quantitative RT-PCR analysis of *DDX21* mRNA levels in cells mock-infected or infected with PRV WT, ΔVP22, or ΔVP22R at an MOI of 0.1 for 24hours. *18S rRNA* was used as an internal control. Data are presented as fold change relative to the mock group. (C) Quantitative RT-PCR analysis of *DDX21* mRNA expression in HEK-293T cells transfected with increasing amounts of Flag-VP22 plasmid. *18S rRNA* was used for normalization. Data are representative of at least three independent experiments with similar results (mean ± SD of n = 3 biological replicates). ** P < 0.01.
(TIF)

S8 Fig (A) HEK-293T cells seeded in 6-cm dishes were transfected with Flag-DDX21 along with the indicated plasmids (HA-GFP or HA-cGAS). At 24 hours post-transfection (hpt), cells were subjected to immunoprecipitation (IP) using anti-Flag magnetic beads. Whole-cell lysates (WCLs) and immunoprecipitated proteins were analyzed by immunoblotting with antibodies against HA, Flag, and β-actin. (B) HEK-293T cells seeded in 6-cm dishes were transfected with HA-DDX21 along with the indicated plasmids (Flag-GFP or Flag-cGAS). At 24 hpt, cells were processed for IP using anti-HA magnetic beads. WCLs and immunoprecipitated proteins were analyzed as described in panel A. (C) PK-15 cells were mock-infected or infected with PRV-WT-GFP, ΔVP22-GFP, or ΔVP22R-GFP at an MOI of 1 for 9 h, Cells were then harvested for measurement of virus DNA copies by RT-PCR. (D) After MG132 (10μM) treatment, PK-15 cells were mock-infected or infected with PRV-WT. Cells were then collected, and either DNase or PBS was added for immunoprecipitation (IP) experiments using anti-DDX21antibody. Whole-cell lysates (WCLs) and immunoprecipitated proteins were analyzed by immunoblotting with antibodies against cGAS, DDX21, VP22 and β-actin. Data are representative of at least three independent experiments with similar results (mean ± SD of n = 3 biological replicates). ** P < 0.01.
(TIF)

S1 Table Sequences of primers used for RT-PCR and plasmid construction, as well as sgRNAs and siRNAs used in this study. (XLSX)

S2 Table Mass spectrometry data presentation in this study. (XLSX)

S3 Table Numerical data of this study. Excel spreadsheet containing the underlying numerical data for Figs 1A, 1B, 1C, 2D, 2E, 2F, 3C, 4A, 4B, 4C, 4D, 4E, 5A, 5C, 5D, 5G, 6J, 7D, 7F, S1A, S1B, S2B, S5A, S5B, S6A, S6B, S7A, S7B, S7C, and S8C in separate sheets.
(XLSX)
