# Supplementary figures and images for: The tegument protein VP22 of pseudorabies virus inhibits cGAS condensation by inducing nuclear-to-cytoplasmic translocation of DDX21
Source: PLoS Pathog. 2025 Sep 29;21(9):e1013549. doi: 10.1371/journal.ppat.1013549 (PMC12503277; doi:10.1371/journal.ppat.1013549)

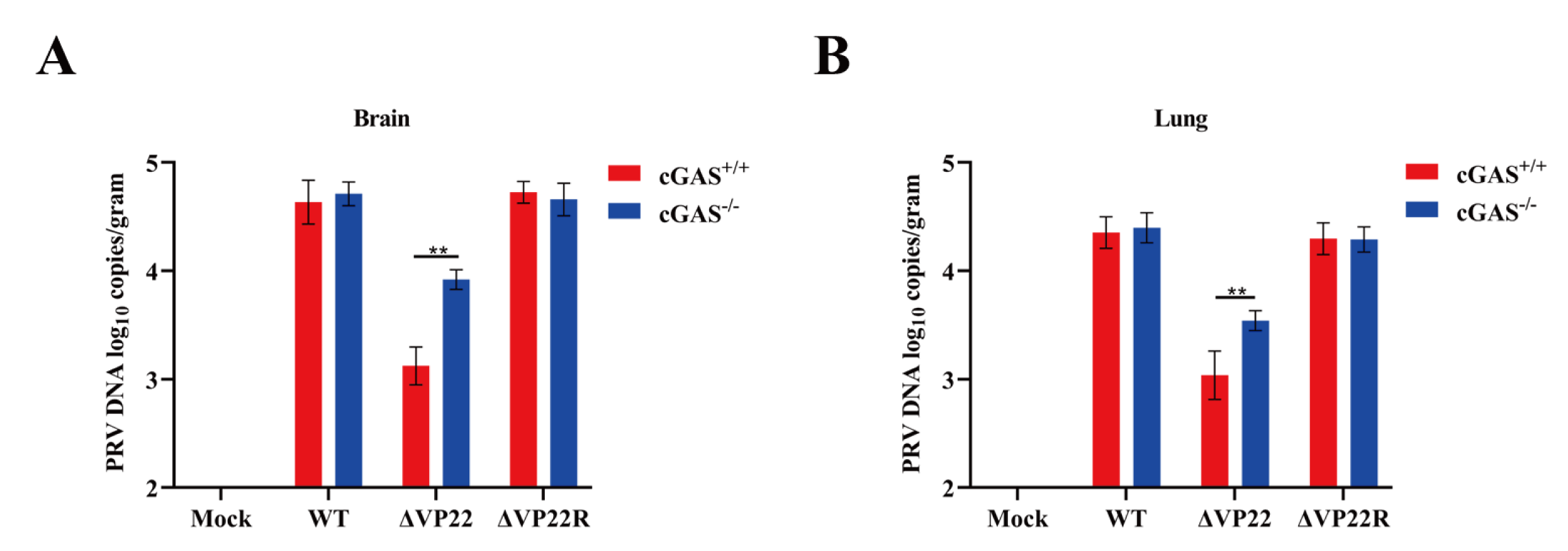

Supplement: S1 Fig — (A)WT and cGAS ⁻ / ⁻ mice (n = 3 per group) were mock-infected or intraperitoneally injected with 1 × 10⁴ PFU of PRV-WT, ΔVP22, or ΔVP22R. At 3 days post-infection (dpi), brains were harvested, and total virus DNA loads were measured by RT PCR. (B) virus DNA loads in lung tissues from the same mice described in panel A. Data represent at least three independent experiments with similar results (mean ± SD, n = 3 biological replicates). ** P < 0.01. (TIF) [file ppat.1013549.s001.tif]

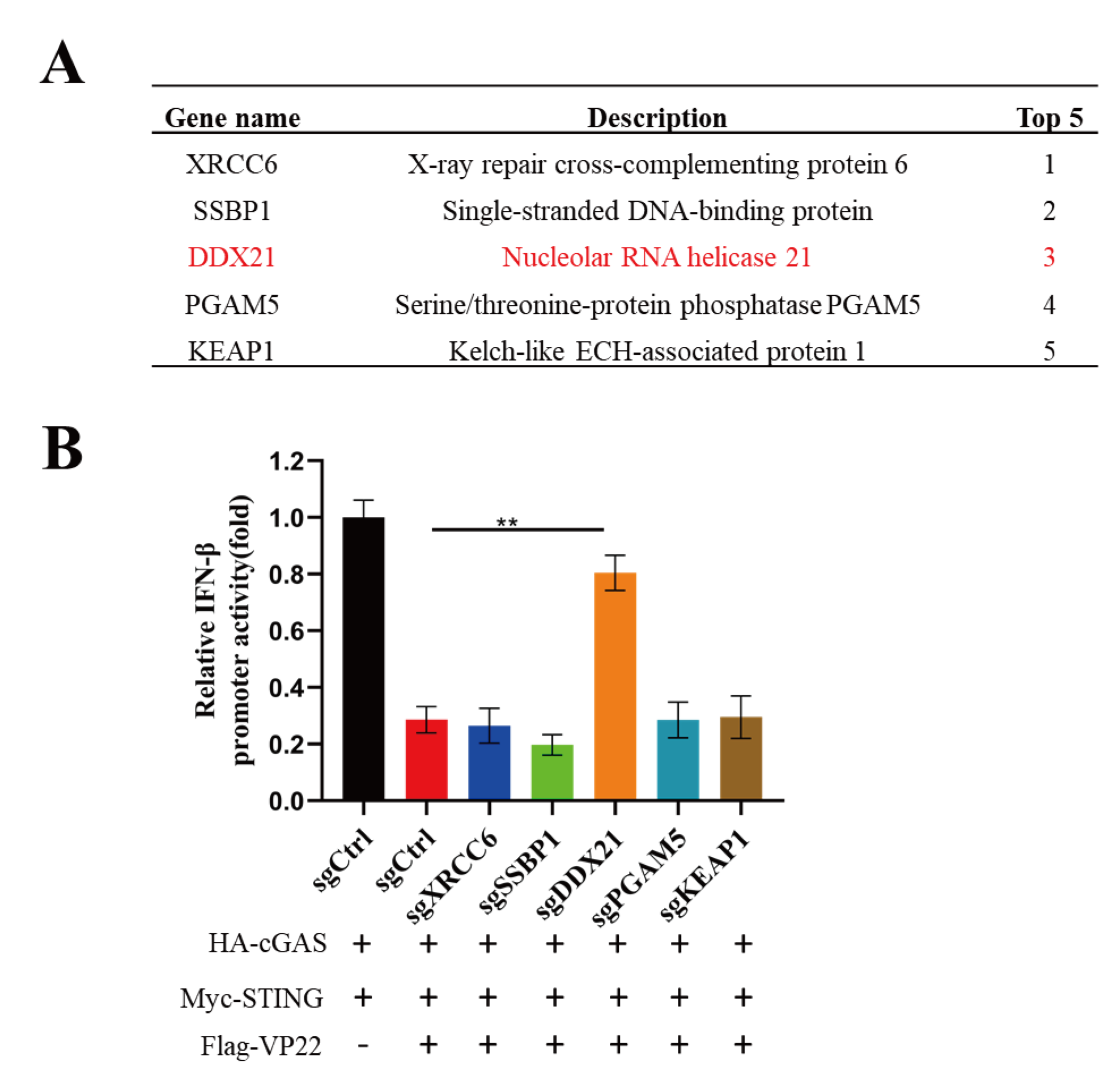

Supplement: S2 Fig — (A) Mass spectrometry (MS) analysis of proteins co-immunoprecipitated with Flag-VP22 from HEK-293T cells. The top five candidate VP22-interacting proteins are listed, ranked by abundance. DDX21 (highlighted in red), a nucleolar RNA helicase, was identified as a top interactor (ranked third). (B) HEK-293T cells with indicated gene knockouts were seeded in 24-well plates and co-transfected with IFN-β-Luc, pRL-TK, HA-cGAS, or Myc-STING, together with Flag-VP22. At 24 h post-transfection (hpt), cells were lysed for luciferase reporter assays. Data represent at least three independent experiments with similar results (mean ± SD, n = 3 biological replicates). ** P < 0.01. (TIF) [file ppat.1013549.s002.tif]

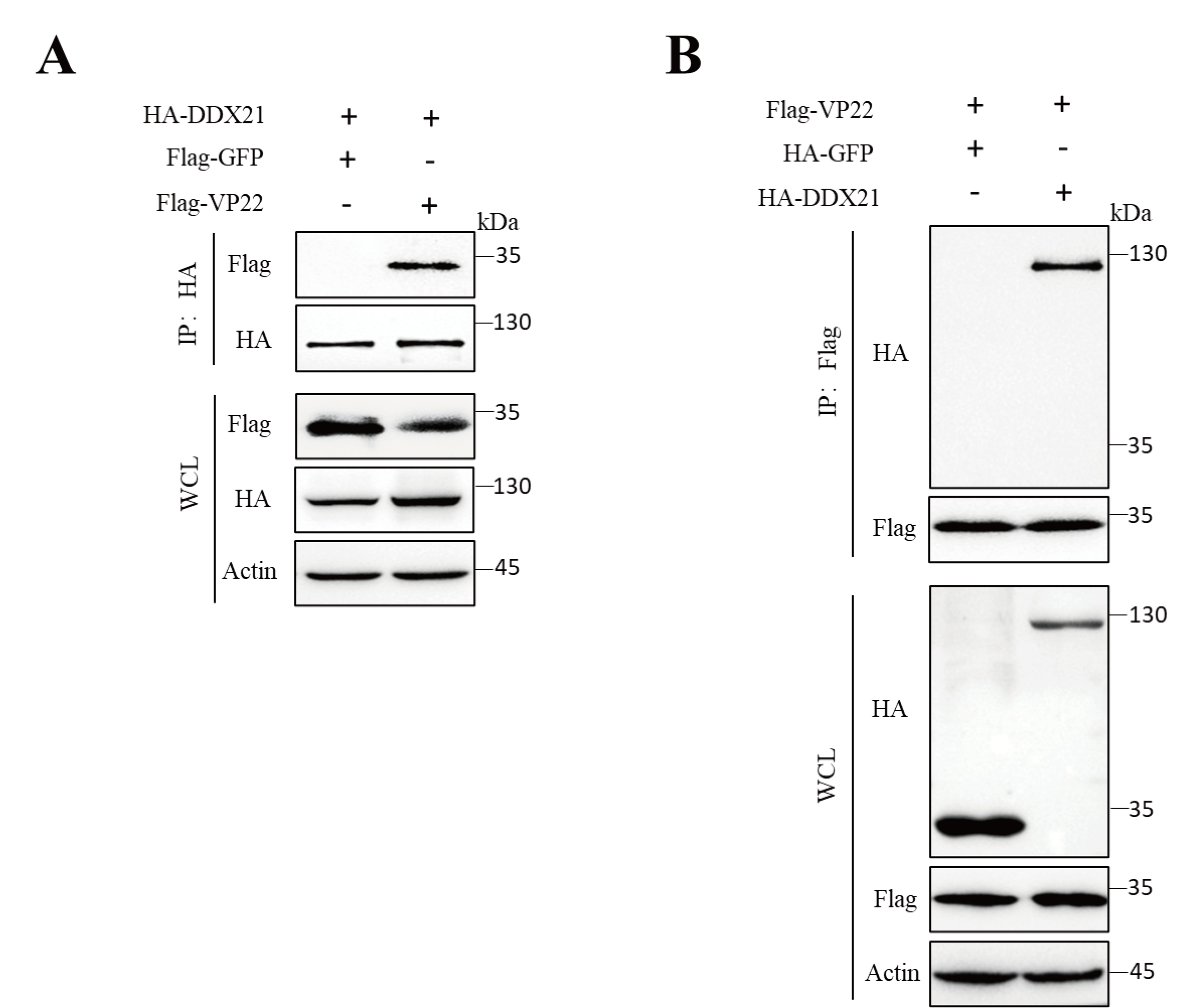

Supplement: S3 Fig — (A) HEK-293T cells were co-transfected with Flag-VP22 and either HA-GFP or HA-DDX21. At 24 h post-transfection (hpt), cell lysates were immunoprecipitated (IP) with anti-Flag beads, and both whole-cell lysates (WCLs) and precipitates were analyzed by immunoblotting with anti-HA, anti-Flag, and anti-β-actin antibodies. (B) HEK-293T cells were co-transfected with HA-DDX21 and either Flag-GFP or Flag-VP22. At 24 hpt, lysates were immunoprecipitated with anti-HA beads and analyzed as in panel A. Data are representative of three independent experiments. (TIF) [file ppat.1013549.s003.tif]

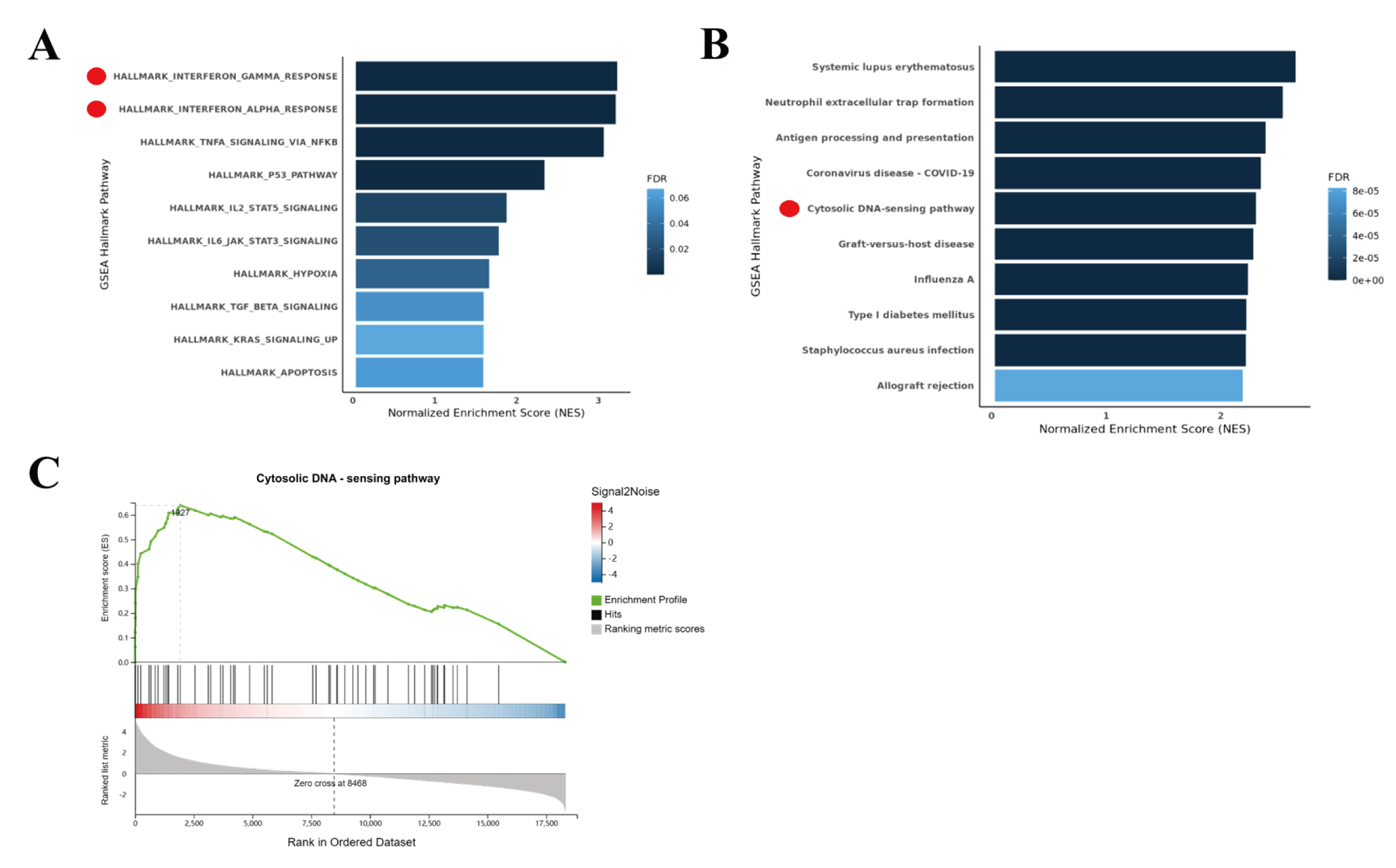

Supplement: S4 Fig — (A) Single-gene GSEA of DDX21-associated genes indicates activation of the Interferon Gamma and Interferon Alpha response pathways. (B) KEGG pathway GSEA based on RNA-seq data from PRV-infected versus mock-treated groups highlights enrichment of the cytosolic DNA-sensing pathway. Bar heights represent NES values, and colors indicate FDR values. (C) GSEA of differentially expressed genes between PRV-infected and mock-treated groups shows significant enrichment of the cytosolic DNA-sensing pathway. (TIF) [file ppat.1013549.s004.tif]

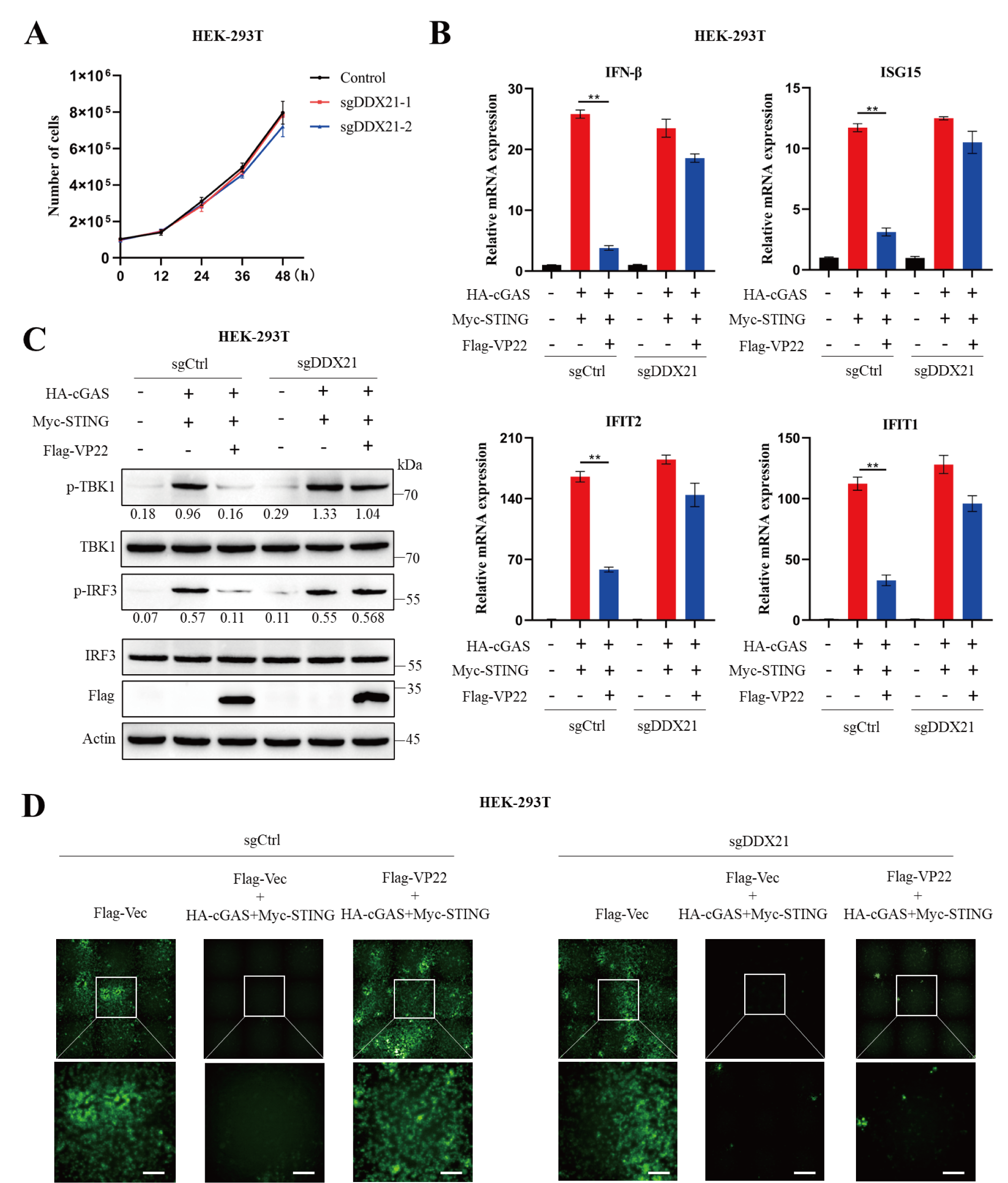

Supplement: S5 Fig — (A) HEK-293T cells were transduced with sgRNAs targeting DDX21 (sg-1, sg-2) or a non-targeting control (sgCtrl). Cell numbers were then counted at 0, 12-, 24-, 36-, and 48-hours post-inoculation. (B) Wild-type and DDX21-knockout HEK-293T cells were transfected with HA-cGAS and Myc-STING along with either Flag-vector or Flag-VP22. After 24 hours, total RNA was extracted and the mRNA levels of IFN-β, ISG15, IFIT1, and IFIT2 were measured by RT-qPCR. Gene expression was normalized to 18S rRNA. (C) Wild-type and DDX21-knockout HEK-293T cells were transfected with HA-cGAS, Myc-STING, and Flag-VP22 for 24 hours. Cell lysates were subjected to western blot analysis using antibodies against Flag, IRF3, phosphorylated IRF3 (p-IRF3), TBK1, phosphorylated TBK1 (p-TBK1), and β-actin. (D) Immunofluorescence analysis of GFP signal in wild-type and DDX21-knockout HEK-293T cells treated as in panel B. Supernatants were transferred to fresh HEK-293T cells, which were then infected with VSV-GFP at an MOI of 0.01. GFP fluorescence was detected 24 hours post-infection. Scale bars: 100 μm. Data are representative of at least three independent experiments with similar results (mean ± SD, n = 3 biological replicates in A and B). ** P < 0.01. (TIF) [file ppat.1013549.s005.tif]

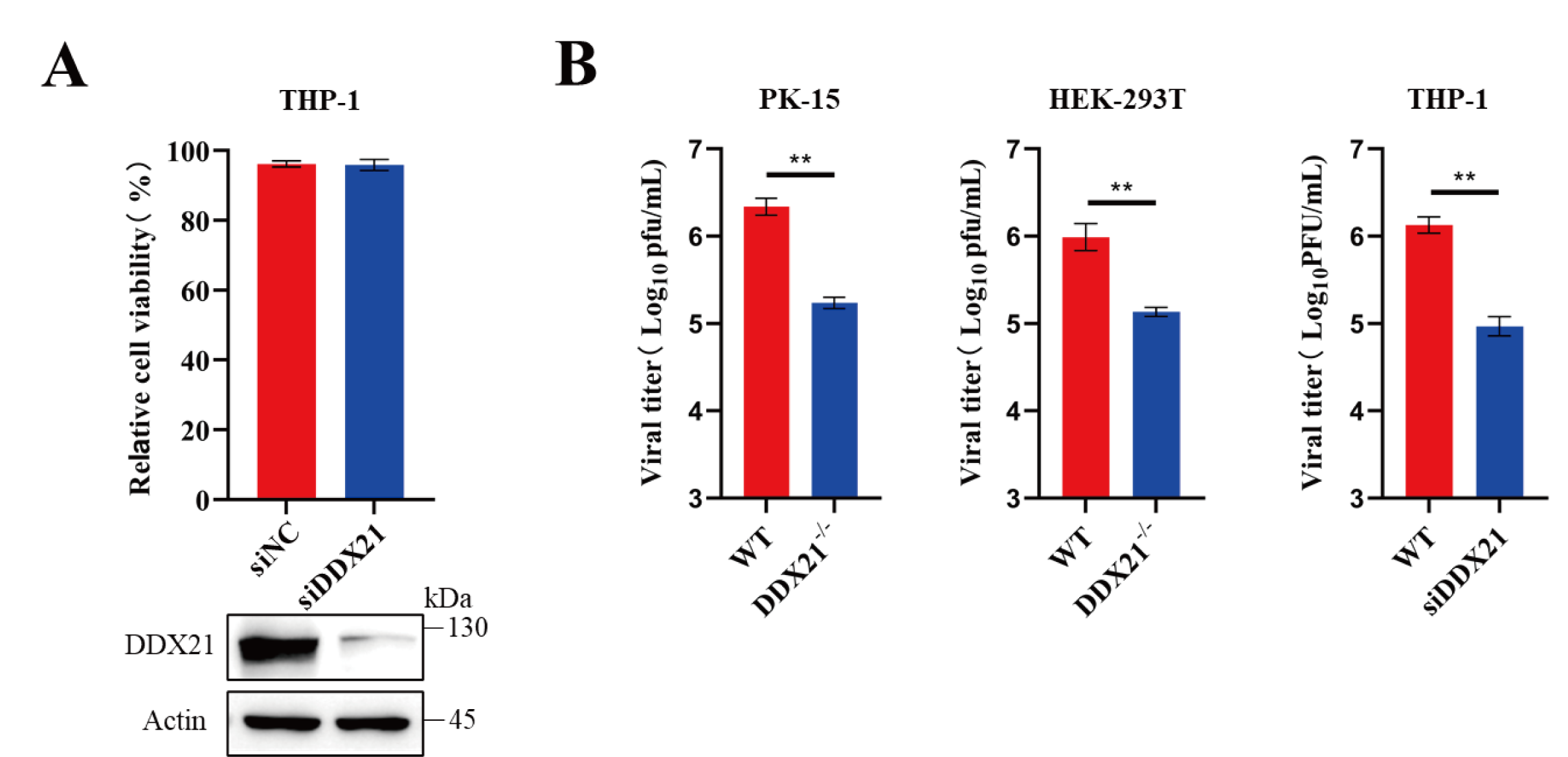

Supplement: S6 Fig — (A) THP-1 cells were transfected with siRNAs targeting DDX21 (si#1) or a non-targeting control (siNC). After 48 hours, cell viability was assessed using a CCK-8 assay (top), and knockdown efficiency was evaluated by immunoblotting (bottom) with antibodies against DDX21 and β-actin. (B) Wild-type and DDX21 ⁻ / ⁻ PK-15 and HEK-293T cells were infected with PRV at an MOI of 0.01 for 48 hours. Separately, THP-1 cells transfected with siDDX21 or siCtrl for 48 hours were also infected with PRV at an MOI of 0.01 for 48 hours. Viral titers in the supernatants were measured by plaque assay. Data are presented as mean ± SD of n = 3 biological replicates. ** P < 0.01. (TIF) [file ppat.1013549.s006.tif]

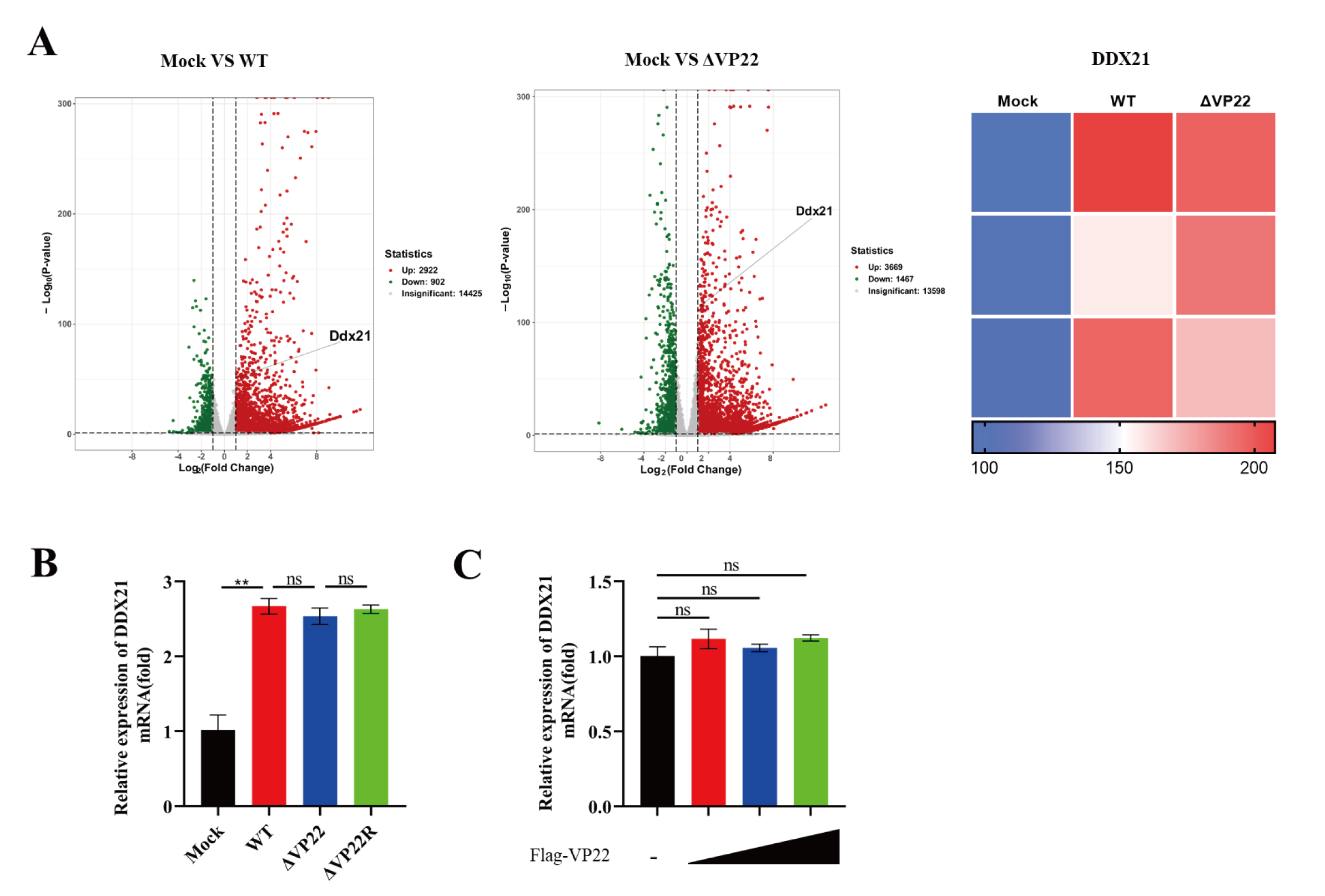

Supplement: S7 Fig — (A) Volcano plots showing differentially expressed genes comparing mock-infected vs. PRV WT (left) and mock-infected vs. PRV ΔVP22 (middle). The heatmap (right) displays the relative expression levels of DDX21 among mock, WT, and ΔVP22 groups. (B) Quantitative RT-PCR analysis of DDX21 mRNA levels in cells mock-infected or infected with PRV WT, ΔVP22, or ΔVP22R at an MOI of 0.1 for 24hours. 18S rRNA was used as an internal control. Data are presented as fold change relative to the mock group. (C) Quantitative RT-PCR analysis of DDX21 mRNA expression in HEK-293T cells transfected with increasing amounts of Flag-VP22 plasmid. 18S rRNA was used for normalization. Data are representative of at least three independent experiments with similar results (mean ± SD of n = 3 biological replicates). ** P < 0.01. (TIF) [file ppat.1013549.s007.tif]

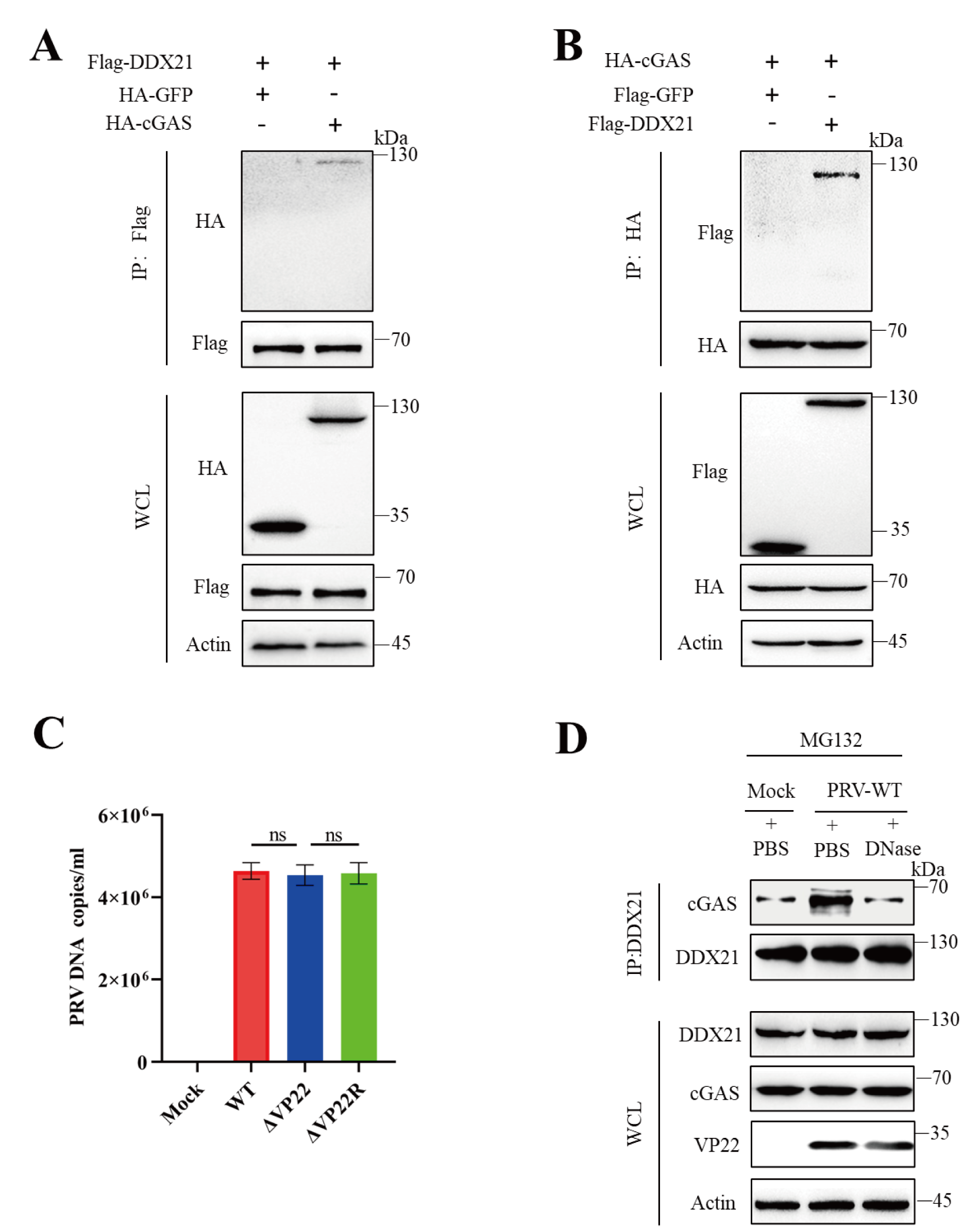

Supplement: S8 Fig — At 24 hours post-transfection (hpt), cells were subjected to immunoprecipitation (IP) using anti-Flag magnetic beads. Whole-cell lysates (WCLs) and immunoprecipitated proteins were analyzed by immunoblotting with antibodies against HA, Flag, and β-actin. (B) HEK-293T cells seeded in 6-cm dishes were transfected with HA-DDX21 along with the indicated plasmids (Flag-GFP or Flag-cGAS). At 24 hpt, cells were processed for IP using anti-HA magnetic beads. WCLs and immunoprecipitated proteins were analyzed as described in panel A. (C) PK-15 cells were mock-infected or infected with PRV-WT-GFP, ΔVP22-GFP, or ΔVP22R-GFP at an MOI of 1 for 9 h, Cells were then harvested for measurement of virus DNA copies by RT-PCR. (D) After MG132 (10μM) treatment, PK-15 cells were mock-infected or infected with PRV-WT. Cells were then collected, and either DNase or PBS was added for immunoprecipitation (IP) experiments using anti-DDX21antibody. Whole-cell lysates (WCLs) and immunoprecipitated proteins were analyzed by immunoblotting with antibodies against cGAS, DDX21, VP22 and β-actin. Data are representative of at least three independent experiments with similar results (mean ± SD of n = 3 biological replicates). ** P < 0.01. (TIF) [file ppat.1013549.s008.tif]
